# Audiovisual spatial recalibration but not integration is shaped by early sensory experience

**DOI:** 10.1016/j.isci.2022.104439

**Published:** 2022-05-23

**Authors:** Patrick Bruns, Lux Li, Maria J.S. Guerreiro, Idris Shareef, Siddhart S. Rajendran, Kabilan Pitchaimuthu, Ramesh Kekunnaya, Brigitte Röder

**Affiliations:** 1 Biological Psychology and Neuropsychology, University of Hamburg, 20146 Hamburg, Germany; 2 Department of Epidemiology and Biostatistics, Schulich School of Medicine & Dentistry, Western University, London, ON N6G 2M1, Canada; 3 Biological Psychology, Department of Psychology, School of Medicine and Health Sciences, University of Oldenburg, 26111 Oldenburg, Germany; 4 Jasti V Ramanamma Children’s Eye Care Centre, LV Prasad Eye Institute, Hyderabad, Telangana 500034, India

**Keywords:** Ophthalmology, Developmental neuroscience, Sensory neuroscience, Cognitive neuroscience

## Abstract

To clarify the role of sensory experience during early development for adult multisensory learning capabilities, we probed audiovisual spatial processing in human individuals who had been born blind because of dense congenital cataracts (CCs) and who subsequently had received cataract removal surgery, some not before adolescence or adulthood. Their ability to integrate audio-visual input and to recalibrate multisensory spatial representations was compared to normally sighted control participants and individuals with a history of developmental (later onset) cataracts. Results in CC individuals revealed both normal multisensory integration in audiovisual trials (ventriloquism effect) and normal recalibration of unimodal auditory localization following audiovisual discrepant exposure (ventriloquism aftereffect) as observed in the control groups. In addition, only the CC group recalibrated unimodal visual localization after audiovisual exposure. Thus, in parallel to typical multisensory integration and learning, atypical crossmodal mechanisms coexisted in CC individuals, suggesting that multisensory recalibration capabilities are defined during a sensitive period in development.

## Highlights

• Audiovisual spatial processing recovers after congenital cataract removal surgery
• Long durations of congenital blindness do not prevent recovery
• Sight-recovery individuals use audiovisual input to recalibrate visual localization
• Typical and atypical crossmodal mechanisms coexist after sight recovery

## Graphical abstract

**Figure:**
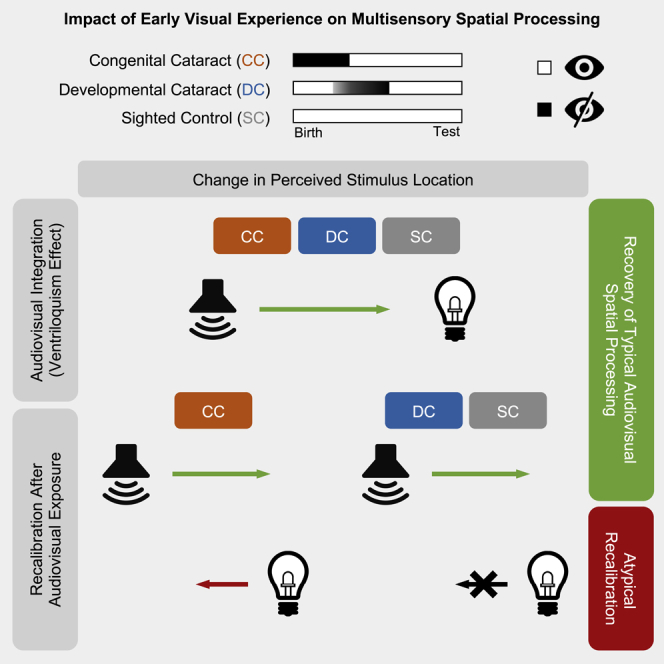

## Introduction

Sensory experiences made during sensitive periods of development have a strong and lasting impact on how information from the different sensory systems is combined (Putzar et al., 2007; Schorr et al., 2005; Smyre et al., 2021; Sourav et al., 2019; Wallace and Stein, 2007). For example, neuroanatomical and neurophysiological studies in owls have revealed distinct neural mechanisms for crossmodal recalibration depending on the age of the animals: Although crossmodal recalibration in juvenile owls was mediated by anatomical changes (DeBello et al., 2001; Feldman and Knudsen, 1997), it was associated with top-down guided physiological changes in adult animals (Hyde and Knudsen, 2002; Knudsen, 1998).

These different neural mechanisms of crossmodal learning in the developing and adult system were impressively demonstrated in an experiment in which juvenile owls were exposed to a short phase of prism experience, resulting in a shift of perceived auditory locations to correct for the visual spatial disparity, and consecutively grew up with typical visual input. When the prisms were refitted in adulthood these animals demonstrated, unlike adult owls without any prism experience early in life, juvenile-like recalibration of auditory spatial maps. Crucially, this prevailing learning capacity was limited to the same prism-mediated shift of the visual world that was experienced during development (Knudsen, 1998; Linkenhoker et al., 2005). These results, hence, suggest that the involved neural systems were not more plastic in general, but that these animals had access to specific additional anatomical connections which had been established during the juvenile prism experience. Thus, it seems that anatomical connectivity acquired during the sensitive period sets the limits of functional adaptation in adulthood and that the maintenance of these connections is at least partially independent of use following the sensitive period.

Behavioral findings in humans with late-onset blindness provide support for this hypothesis as well. Despite dramatic changes in the accessible sensory world, late blind humans have been shown to use the typical visually defined spatial reference frame for representing touch in tasks in which congenitally blind humans use somatotopically defined (Collignon et al., 2009; Röder et al., 2004) or head-centered references frames (Röder et al., 2007), even if blindness had lasted for more years than in the congenitally blind participants of the same study. Similarly, it has been reported that congenitally blind humans lack typical sound-shape associations observed in sighted participants (Fryer et al., 2014; Hamilton-Fletcher et al., 2018; Sourav et al., 2019), whereas late blind humans still demonstrate them many years after total blindness had started (Sourav et al., 2019).

If crossmodal connectivity is indeed acquired during an early sensitive phase of development and stabilized in a way that prevents loss later in life, individuals who were born blind but recovered sight later in life should demonstrate altered multisensory processes reminiscent of those observed in congenitally blind humans. Moreover, multisensory interactions of the newly acquired visual system with the auditory and tactile systems would be expected to differ. The evidence currently available draws an inconsistent picture: Individuals who had been born blind because of bilateral dense cataracts and who had undergone cataract removal surgery later in life demonstrated processing gains from redundant simple crossmodal stimulation (De Heering et al., 2016; Putzar et al., 2007, 2012) and even showed visual-haptic integration as assessed by the precision of size estimates (Senna et al., 2021) or the occurrence of the size-weight illusion (Pant et al., 2021). However, they were less able to integrate audio-visual speech input (Putzar et al., 2007, 2010), did not show typical crossmodal sound-shape associations (Sourav et al., 2019), and used spatial reference frames typical for congenitally blind humans (Azanon et al., 2018; Ley et al., 2013; cf. Collignon et al., 2009; Röder et al., 2004).

Altered multisensory processing might be related to impaired multisensory learning mechanisms such as crossmodal recalibration. Recent findings have suggested two dissociable types of crossmodal recalibration which emerge at two distinct time scales: (1) Immediate recalibration on a trial-by-trial basis following a single exposure to a discrepant crossmodal stimulus and (2) cumulative recalibration after prolonged exposure to a consistent crossmodal discrepancy (Bruns and Röder, 2015; Park and Kayser, 2021; Watson et al., 2019). However, whether or how immediate and cumulative multisensory learning mechanisms depend on early sensory input has not been investigated in humans yet. A study in children has recently found that crossmodal spatial recalibration does not arise before middle childhood (with immediate recalibration occurring before cumulative recalibration) and, thus, develops later than the ability to integrate auditory and visual spatial input for localization (Rohlf et al., 2020). Moreover, it has been suggested that the weights assigned to each sensory cue during cumulative recalibration in adulthood do not depend on their precision but rather seem to be fixed (Rohlf et al., 2021; Zaidel et al., 2011; but see Hong et al., 2021), whereas they are dependent on cue reliability in both immediate recalibration and multisensory integration (Rohlf et al., 2021). Thus, the late developmental onset of cumulative crossmodal recalibration might be a consequence of the need to acquire the relative weighting of individual sensory cues through extensive experience which is consecutively stabilized by an elaboration of anatomical connectivity during development (Linkenhoker et al., 2005).

Multisensory integration requires not only a proper weighting of the sensory cues but also a decision of whether the sensory cues arise from a common cause and, thus, should be integrated at all (Kayser and Shams, 2015). Humans seem to solve this causal inference problem implicitly and optimally (or near-optimally) as predicted by Bayesian hierarchical causal inference (CI) models (Körding et al., 2007; Wozny et al., 2010), which account for both the sensory cue binding and weighting aspects by assuming that two perceptual estimates are derived, one under the assumption that the sensory cues originated from a single source (fusion) and one under the assumption that they had separate sources (segregation). Previous studies have suggested some variability across individuals in the decision strategy that is used to integrate these two estimates (Wozny et al., 2010): The majority of participants selected one of the two causal structures in proportion to their probability (probability matching), but in some participants the behavioral responses were better described by a weighted averaging of the two estimates (model averaging) or by the selection of the most likely causal structure (model selection). Regardless of the specific individual decision strategy used, the superiority of the CI models in explaining human behavior, as compared to a model that only takes cue reliability but not the causal structure into account (forced fusion models), suggests that multisensory integration typically incorporates both causal inference and cue reliability.

If early crossmodal experience is critical for multisensory spatial integration and recalibration capabilities to emerge, we would expect altered multisensory spatial processing in CC reversal individuals because of the lack of visual (and as a consequence altered crossmodal) input after birth. The present study tested this hypothesis by investigating both multisensory integration and crossmodal recalibration in individuals who had been born blind because of bilateral dense cataracts which were removed between the ages of 5 months and 33 years. Although congenital bilateral dense cataracts typically do not result in total blindness (i.e., a loss of light perception), the CC reversal individuals selected for the present study fulfilled the criteria of blindness according to the classification of the World Health Organization (2019) before cataract-removal surgery (i.e., visual acuity worse than 3/60).

**Figure 1:**
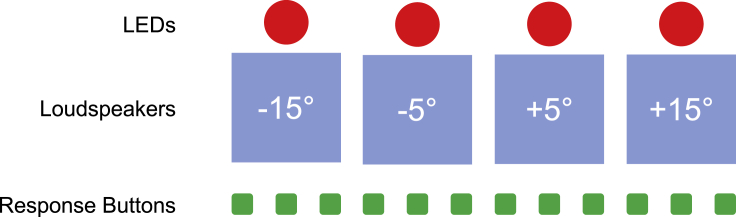
Schematic illustration of the experimental setup Participants faced the center of four loudspeakers positioned at ±15° and ±5°. Red LEDs were attached on top of each loudspeaker. An array of 12 pushbuttons, which had the same width as the loudspeaker array, was placed in front of the loudspeakers.

We used a typical and well-established ventriloquism paradigm with simple sounds and visual stimuli which were presented with a spatial disparity. The participants’ task was to localize both the auditory and the visual component of the crossmodal stimuli, which allowed us to assess the ability to spatially integrate audiovisual input after reversal of a congenital blindness. Additional unimodal visual and auditory test trials were included to assess the immediate (VAEi) and cumulative ventriloquism aftereffects (VAEc) indicating crossmodal recalibration (for a schematic illustration of the experimental setup, see Figure 1). We expected a smaller ventriloquism effect (VE), and relatedly a smaller VAEi, because of prevailing visual deficits in CC reversal individuals compared to normally sighted controls. If normative causal inference and optimal multisensory integration require exposure to audiovisual correspondences during a sensitive phase of early development, we would also expect the localization performance of CC reversal individuals to be less well described by a CI model. Moreover, if the relative weighting of individual cues for cumulative crossmodal recalibration is acquired during a sensitive phase of development, we would expect a lower influence of vision on the recalibration of auditory spatial representations but a higher influence of hearing on recalibration of visual spatial representations in CC reversal individuals compared to normally sighted controls and individuals who had been treated for developmental (i.e., late onset) cataracts.

## Results

To determine the influence of early visual experience on multisensory integration (as indicated by the VE) and learning (as indicated by the VAE) in later life, we compared a group of 11 individuals who had been born blind because of dense bilateral congenital cataracts (CC group), and who underwent cataract-removal surgeries later in life (between 5 months and 33 years of age), with two control groups, one group of 10 normally sighted control participants (SC group) and one group of 10 individuals with developmental cataracts (DC group). All participants had to localize auditory (A) and visual (V) stimuli coming from four different azimuthal positions. A and V stimuli were either presented together with different audiovisual (AV) spatial discrepancies (adaptation trials used to assess the VE) or were presented alone as unimodal test trials to assess immediate recalibration effects (VAEi), that is, a shift in sound or visual localization after a single exposure to a spatially discrepant AV stimulus. In a second block, the AV spatial discrepancy was fixed at 10° to assess cumulative recalibration effects (VAEc) in the unimodal test trials.

### Early visual input is not necessary for the development of audiovisual spatial integration (VE)

**Figure 2:**
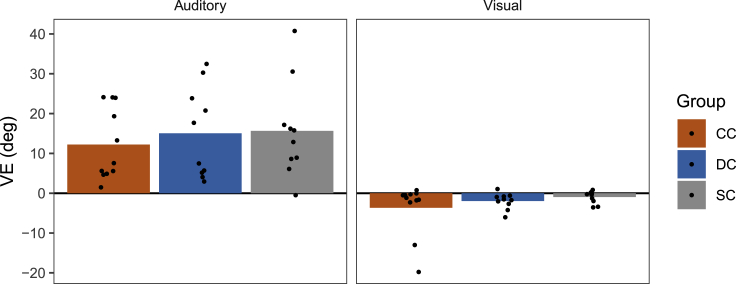
Ventriloquism effects (VE) in congenital cataract (CC), developmental cataract (DC), and sighted control (SC) individuals The differences in localization responses (in degrees) between audiovisual (AV) trials with a rightward spatial discrepancy (V to the right of A) and AV trials with a leftward discrepancy (V to the left of A) are shown separately for each group and response modality. Bars indicate group averages and dots indicate individual values. Positive values indicate localization biases toward the V location and negative values indicate localization biases toward the A location. (See also Figures S1–S4, S7, and S8).

In AV trials, participants had to localize both the A and the V stimulus components as in previous studies of the VE (Jackson, 1953; Körding et al., 2007; Mohl et al., 2020; Wozny et al., 2010; Wozny and Shams, 2011). The degree to which these responses were modulated by the AV spatial discrepancy (i.e., the size of the VE) is, thus, an indicator of multisensory integration. Here we defined the VE as the difference in localization responses between AV trials with a rightward discrepancy (V to the right from location A) and AV trials with a leftward discrepancy (V to the left from location A). The resulting VE values are shown in Figure 2 (for group-averaged responses as a function of AV discrepancy, see also Figure S1).

In all three groups, A responses were clearly biased toward the V location [p≤0.002; BF_+0_≥ 36.21], indicative of an auditory VE. The size of the auditory VE did not significantly differ between groups [*F*(2,17.90) = 0.33, p = 0.720; BF_10_ = 0.25]. Conversely, V responses tended to be biased toward the A location. As for the auditory VE, there was no significant difference in the size of the visual VE between groups [*F*(2,17.11) = 1.39, p = 0.276; BF_10_ = 0.43], although the visual VE only reached significance in the DC group [p = 0.019, BF_-0_ = 10.04] and not in the CC and SC groups [p≥0.080, BF_-0_≤ 2.35]. Thus, all three groups exhibited similar multisensory integration capabilities in the AV trials of Block 1.

To further characterize multisensory integration in all groups, we next modeled the spatial distributions of A and V responses as a function of the AV discrepancy (see Figures S2–S5). We fitted and compared three Bayesian hierarchical causal inference (CI) models of multisensory perception, each with a different decision strategy (Wozny et al., 2010): model averaging (CI-MA), probability matching (CI-PM), and model selection (CI-MS), and four non-CI baseline models to each participant’s Block 1 AV localization responses (ventriloquism effect). The CI models accounted for uncertainty in the causal structure that could give rise to the A and V cues, that is, the relative probability of the same versus different sources causing the cues was assessed to determine whether and how to integrate the cues (Körding et al., 2007). Unlike the CI models, the baseline models assumed a fixed causal structure and either always integrated (forced fusion, FF) or always segregated (SG) the A and V cues to estimate the stimulus locations. The FF and SG models each had two forms: either incorporating a Gaussian location prior (FF, SG) or no priors (FFnp, SGnp). The best model for an individual participant was selected based on having the lowest Bayesian information criterion (BIC) value (Tables 1 and S1). No participant was best fitted by model FFnp or SGnp. A Chi-square test of independence on the numbers of participants best fitted by the other five models indicated that the distribution of best-fitting models did not depend on the group [$\chi^{2}$(8) = 11.42, p = 0.179].

**Table 1:** Comparison of model performances

	CI-MA	CI-PM	CI-MS	FF	SG	FFnp	SGnp
CC (n = 11)	836.97 ±118.11 (3)	**797.99** **±114.67 (1)**	813.68 ±115.86 (2)	1761.80 ±147.40 (6)	961.10 ±139.48 (4)	1681.48 ±147.35 (7)	985.67 ±137.35 (5)
DC (n = 10)	1010.54 ±83.77 (3)	971.98 ±77.51 (2)	**967.90** **±78.76 (1)**	1808.48 ±166.79 (6)	1051.49 ±97.44 (4)	1814.98 ±167.54 (7)	1061.66 ±97.42 (5)
SC (n = 10)	913.16 ±116.04 (3)	**880.39** **±110.71 (1)**	892.67 ±107.29 (2)	1859.86 ±266.33 (6)	1031.62 ±120.40 (4)	1875.26 ±262.58 (7)	1059.65 ±116.43 (5)

The table shows the mean ± SEM Bayesian information criterion (BIC) values of model fits to Block 1 audiovisual localization data across individually fitted participants. A lower BIC indicates better model performance for a given dataset. Bracketed numbers indicate each model’s ranking within a group. Boldfaced is the overall best model within each group. According to Raftery (1995), a BIC difference of 0–2 between two models suggests weak evidence as to which model is better, a BIC difference of 2-6 shows positive evidence (in favor of the model with the lower BIC), a BIC difference of 6–10 shows strong evidence, and a BIC difference >10 shows very strong evidence. Being compared are three causal inference models (CI-MA: causal inference with model averaging; CI-PM: causal inference with probability matching; CI-MS: causal inference with model selection) and four baseline models (FF: forced fusion with location prior; SG: segregation with location prior; FFnp: forced fusion with no priors; SGnp: segregation with no priors). (See also Tables S1 and S2 and Figures S2–S5).

Overall, model CI-PM predicted the AV localization responses best, as it was the best-fitting model for 17 of 31 (54.8%) participants in the total sample including the majority of CC and SC participants (Table S1), and had the lowest overall BICs for CC and SC participants (Table 1). CI-MS accounted for most of the remaining participants (22.6%) and was the best-fitting model in DC participants, with CI-PM performing only slightly worse than CI-MS in this group (Table 1). Overall, this finding is similar to previous behavioral studies of the VE in normally sighted human participants, which found that CI-PM explained the AV spatial localization data better than CI-MA and CI-MS (Wozny et al., 2010; Wozny and Shams, 2011). Importantly, in all three groups the BIC values indicated that all three CI models described the data substantially better than each of the four non-CI models. The forced fusion model with no priors (FFnp), commonly known as the optimal integration model (Alais and Burr, 2004; Ernst and Banks, 2002), described the data worst and did not improve much even after incorporating the location prior (Table 1). In summary, the observed AV localization pattern of most participants, including the majority of CC participants, was highly consistent with Bayesian causal inference, as evident by the overall superior performance of the CI models compared with the baseline models. These findings suggest that multisensory integration in CC individuals was guided by similar computational principles as in the control groups.

### Immediate crossmodal recalibration (VAEi) recovers after restoring sight in congenital blindness

**Figure 3:**
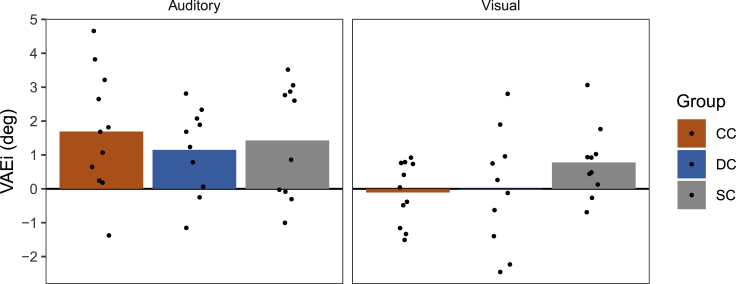
Immediate ventriloquism aftereffects (VAEi) in CC, DC, and SC individuals The differences in localization responses (in degrees) between unimodal trials (A and V trials, respectively) which were preceded by an AV trial with a rightward discrepancy (V to the right of A) and unimodal trials which were preceded by an AV trial with a leftward discrepancy (V to the left of A) are shown separately for each group and modality. Bars indicate group averages and dots indicate individual values. Positive values indicate localization biases toward the V location and negative values indicate localization biases toward the A location. (See also Figures S6–S8).

To assess whether participants immediately recalibrated their unimodal localization in response to spatially discrepant AV stimuli on a trial-by-trial basis, we computed the difference in localization responses between unimodal trials (A and V trials, respectively) which were preceded by an AV trial with a rightward discrepancy and unimodal trials which were preceded by an AV trial with a leftward discrepancy. The resulting VAEi values are shown in Figure 3 (for group-averaged responses as a function of the spatial discrepancy in the preceding AV trial, see also Figure S6).

In all three groups, localization responses in A trials were biased in the direction of the preceding AV discrepancy [p≤0.018, BF_+0_≥ 5.68], indicating a typical auditory VAEi. The size of the auditory VAEi did not significantly differ between groups [*F*(2,18.34) = 0.32, p = 0.728; BF_10_ = 0.25]. In contrast to the auditory VAEi, localization responses in V trials were not significantly modulated by the preceding AV spatial discrepancy in any of the three groups [p>0.999, BF_-0_≤ 0.41] and there were no significant group differences [*F*(2,17.35) = 2.10, p = 0.153; BF_10_ = 0.54]. Thus, on a trial-by-trial basis, all three groups recalibrated auditory but not visual localization in response to immediately preceding spatially discrepant AV stimuli.

**Figure 4:**
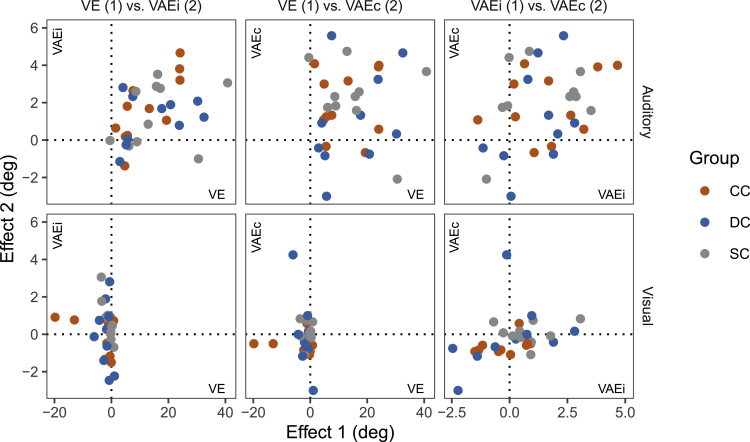
Correlations between ventriloquism effects (VE), immediate (VAEi) and cumulative (VAEc) ventriloquism aftereffects Bracketed numbers indicate which effect is shown on the *x*-axis (Effect 1) and which effect is shown on the *y-*axis (Effect 2) in each subplot. Scatterplots are shown separately for auditory and visual effects. Colors indicate participant groups.

Across groups, the size of the auditory VAEi was significantly correlated with the size of the auditory VE [*r*_s_ = 0.49, p = 0.005], suggesting a link between multisensory integration and immediate crossmodal recalibration (see Figure 4). The correlation between auditory VE and VAEi was significant within the group of CC individuals [p = 0.012], but not within the DC and SC groups [p≥0.494].

### Early sensory experience defines the role of vision and hearing for calibrating multisensory spatial representations (VAEc)

**Figure 5:**
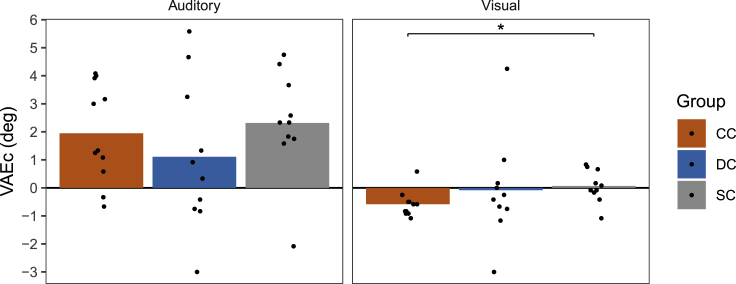
Cumulative ventriloquism aftereffects (VAEc) in CC, DC, and SC individuals The differences in localization responses (in degrees) between unimodal trials (A and V trials, respectively) in Block 2, in which AV trials featured a constant spatial discrepancy of 10°, and unimodal localization responses in Block 1, in which AV trials had a mean spatial discrepancy of 0°, are shown separately for each group and modality. Bars indicate group averages and dots indicate individual values. Positive values indicate localization biases toward the V location and negative values indicate localization biases toward the A location. A Welch ANOVA indicated a significant group difference in the size of the visual VAEc, which was followed up with Games-Howell post-hoc comparisons. Stars denote significant post-hoc comparisons; ∗p<0.05. (See also Figures S2–S4, S7, and S8).

To assess whether participants recalibrated their unimodal localization in response to cumulative evidence for a crossmodal spatial mismatch, we calculated the difference in unimodal localization responses (A and V trials, respectively) between Block 2, in which AV trials featured a constant spatial discrepancy of 10°, and unimodal localization responses in Block 1, in which AV trials had a mean spatial discrepancy of 0°. The resulting VAEc values (see Figure 5) correspond to changes in the likelihood mean bias parameters (${\text{Δ}}_{A}$, ${\text{Δ}}_{V}$) of our computational models from Block 1 to Block 2 (for tests of changes in other model parameters, see Table S2 and Figures S2–S4).

In the CC and SC groups, localization responses in A trials were significantly biased in the direction of the constant AV spatial discrepancy in Block 2 [p≤ 0.006, BF_+0_≥ 24.30], indicating a typical auditory VAEc. The auditory VAEc was not significant in the DC group [p = 0.111, BF_+0_ = 1.06]. Despite this, the size of the auditory VAEc did not significantly differ between groups [*F*(2,17.84) = 0.65, p = 0.532; BF_10_ = 0.36]. CC individuals also showed a highly significant visual VAEc in V trials [p = 0.003; BF_-0_ = 51.33] which was not seen in DC and SC individuals [p≥ 0.890; BF_-0_≤ 0.34]. Accordingly, the size of the visual VAEc significantly differed between groups [*F*(2,16.09) = 3.91, p = 0.041; BF_10_ = 0.39]. Games-Howell post-hoc comparisons confirmed that the visual VAEc in the CC group was significantly larger than in the SC group [p = 0.030], although these two groups did not significantly differ from the DC group [both p≥ 0.691]. Thus, although all three groups recalibrated auditory localization in response to a constant AV spatial discrepancy, only CC individuals additionally recalibrated their visual localization.

The size of the auditory VAEc was neither significantly correlated with the size of the auditory VE [across groups: *r*_s_ = 0.07, p = 0.692; within groups: *r*_s_≤ 0.43, p≥ 0.654] nor with the size of the auditory VAEi [across groups: *r*_s_ = 0.27, p = 0.139; within groups: *r*_s_≤ 0.47, p≥ 0.534]. Similarly, the size of the visual VAEc was not significantly correlated with the size of the visual VE [across groups: *r*_s_ =−0.10, p = 0.613; within groups: *r*_s_≤−0.06, p≥ 0.450] (see Figure 4).

### The duration of blindness may influence the degree to which (but not whether) audiovisual spatial functions recover

Because logMAR (logarithm of the Minimum Angle of Resolution) visual acuity varied between 0.21 and 1.29 in CC individuals and between −0.05 and 0.63 in DC individuals and was considerably worse in both groups compared to SC individuals who all had normal or corrected-to-normal vision, we tested whether visual acuity was correlated with the size of the VE, VAEi, and VAEc effects (see Figure S7). None of the effects were significantly correlated with visual acuity, neither in the CC group [p>0.999], nor in the DC group [p≥0.396].

**Figure 6:**
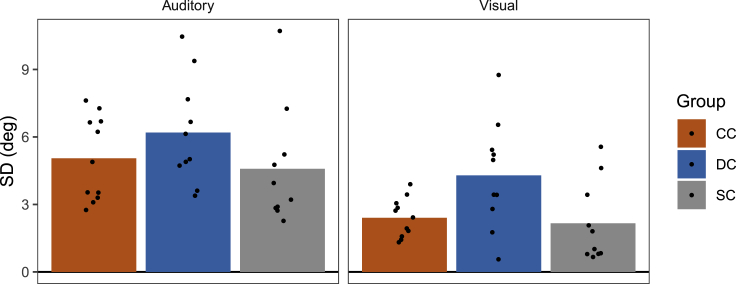
Unimodal localization precision in CC, DC, and SC individuals The standard deviations of the localization responses (in degrees) in A and V trials of Block 1 are shown separately for each group and modality. Bars indicate group averages and dots indicate individual values.

We also verified that all participants were able to reliably localize the A and V stimuli. To this end, we computed localization precision in Block 1 as the individual *SD*s of the localization responses in A and V trials, respectively (see Figure 6). There were no significant group differences in unimodal localization precision between CC, DC, and SC individuals, neither for A precision [*F*(2,17.81) = 1.14, p = 0.344; BF_10_ = 0.48] nor for V precision [*F*(2,15.07) = 2.90, p = 0.086; BF_10_ = 3.00]. In all three groups, V precision was significantly higher than A precision [p≤ 0.025; BF_+0_≥ 3.35]. This suggests that all participants, even those with severe visual impairments, were well able to localize the A and V stimuli used in the present study.

Finally, in an exploratory analysis we compared the sizes of VE, VAEi, and VAEc effects between those CC individuals (n = 4) who had their cataract removed relatively early in life (between 5 and 25 months of age) and those CC individuals (n = 6) who had cataract surgery only much later in life (at or after 14 years of age). The auditory VE, VAEi, and VAEc tended to be weaker and the visual VE and VAEc tended to be stronger in CC individuals who had experienced an extended period of blindness from birth (see Figure S8). However, none of these differences reached statistical significance [p≥ 0.334; BF_10_≤ 0.66].

## Discussion

Although studies in owls have demonstrated a crucial role of crossmodal experience for adult multisensory learning (recalibration) capabilities, it has remained unclear how early visual experience affects human multisensory learning. In a rare human model, sight-recovery individuals with a (in several cases extensive) history of congenital blindness because of bilateral dense cataracts, the present study found surprisingly normal multisensory spatial integration (as indicated by the VE) and recalibration (as indicated by the auditory VAEi and VAEc) capabilities. However, in addition to using visual spatial information to recalibrate auditory localization (auditory VAEc), as seen in normally sighted controls and individuals who had received cataract removal surgery for incomplete or later developed cataracts, only CC reversal individuals used auditory spatial information to recalibrate visual localization (visual VAEc). Both effects, additional use of auditory input to calibrate visual spatial representations (visual VAEc) and intact multisensory integration as assessed by the VE and computational modeling, were consistently seen across CC individuals who all had received cataract-removal surgery late (i.e., at or after the age of 5 months) but whose individual ages at cataract removal surgery varied considerably up to 33 years. Thus, we speculate that the additional recalibration mechanism observed in CC reversal individuals originates from a neural mechanism existing as a consequence of congenital visual deprivation.

Studies in owls (Brainard and Knudsen, 1998; DeBello et al., 2001; Hyde and Knudsen, 2002) that had manipulated the crossmodal spatial correspondence of auditory and visual input through the use of prisms, found both a reduced elimination of anatomical connections during development and an additional axonal growth that served the atypical mapping induced by prism experience (DeBello et al., 2001). These anatomical changes allowed the prism-reared animals as adults to toggle between two parallel and independent neural organizations, a typical and an atypical one, likely by employing inhibitory mechanisms (Hyde and Knudsen, 2002). These findings suggest a more general developmental principle: Experience shapes structural brain networks only during sensitive periods and these neural networks then build the scaffold for future learning. Applied to our human model, in which we compared the effects of presence versus absence of visual input during early development, we speculate that CC individuals had preserved typical multisensory connectivity, allowing them to regain typical multisensory spatial functions, including the VE and the auditory VAEc, after sight restoration. Thus, we suggest that CC individuals were able to “fall back” to a typical organization (Brainard and Knudsen, 1998). In addition, we suggest that they had stabilized an additional set of neural connectivity as a consequence of congenital visual deprivation resulting in the later use of auditory spatial information for calibrating visual representations (visual VAEc). Thus, CC individuals might achieve internal consistency between auditory and visual spatial representations by recalibrating both auditory and visual rather than only auditory spatial representations. A similar coexistence of typical and atypical multisensory links has been observed for the interaction of auditory and visual motion processing (Guerreiro et al., 2016) as well. Although a change of auditory motion perception (a motion aftereffect) after observing visual motion stimuli was found in both sighted controls and CC individuals, a visual after-effect following the presentation of auditory motion was only observed in CC individuals. Thus, in both cases the typical multisensory function characterized by a visual dominance recovered in CC individuals, despite the maintenance of an additional atypical multisensory link.

In congenitally blind humans, evidence for a stronger auditory-driven activity of multisensory parietal cortex than in sighted individuals was reported during auditory localization tasks (Gougoux et al., 2005; Renier et al., 2010; Röder et al., 1999). A stronger auditory influence on multisensory brain regions might either arise from a lack of pruning or a lack of inhibition of exuberant crossmodal connections, as is typically occurring during development (Johannsen and Röder, 2014; see Lewkowicz and Röder, 2012), or from an elaboration of additional connections strengthening the auditory influence in multisensory structures (Rauschecker, 1995). Given the lack of visual information, such a strengthening might be useful for an efficient orienting to and localization of external events. After cataract removal, the additional connections serving auditory spatial input during blindness might (as the atypical connectivity in owls, see Brainard and Knudsen, 1998; DeBello et al., 2001) not be lost but rather be kept and used as a second mechanism for achieving crossmodal consistency. Results from a recent analysis of blood-oxygen-level-dependent (BOLD) resting state activity in CC reversal individuals, most of them with long visual deprivation periods, have indeed suggested altered audio-visual system interactions (Raczy et al., in press). Moreover, previous studies in CC reversal individuals had suggested an enhanced auditory influence in visual cortex after sight restoration (Collignon et al., 2015; Guerreiro et al., 2015). However, how these changes in crossmodal interactions relate to the current behavioral findings remains undissolved.

In contrast to cumulative crossmodal recalibration (VAEc), we did not observe any significant differences in multisensory integration (VE) capabilities between CC individuals and normally sighted controls: CC individuals featured a significant auditory and a nonsignificant visual VE as did the SC group. A strong visual influence on auditory localization in CC individuals fits with their higher visual compared to their auditory localization precision in the present study, as found in the normally sighted controls. Computational modeling analyses indeed suggested an employment of similar multisensory integration principles in CC individuals as typically observed in the normally sighted population (Körding et al., 2007; Wozny et al., 2010) and replicated in the present study in both SC and DC individuals: Causal inference models explained multisensory integration best in all three groups. Consistent with previous studies, there was some variation in the best-fitting CI decision strategy across individuals. Nevertheless, the majority of our participants, including the majority of the CC individuals, were best described by a probability matching strategy (Wozny et al., 2010). Regardless of the variation in individual decision strategies, the superiority of the CI models indicates that the behavioral localization responses were based on a normative causal inference process in all three groups. Thus, in a task that is typically characterized by a strong visual dominance and by a decline of the visual influence over development (Rohlf et al., 2020), we observed extensive recovery in individuals who had experienced a phase of congenital blindness before sight restoration (see also Senna et al., 2021). A similar result pattern emerged for immediate (trial-by-trial) recalibration: Despite an additional visual VAEc in CC individuals, an additional visual VAEi was not detected, whereas a typical auditory VAEi was revealed. In fact, the size of the auditory VAEi and VE was significantly correlated in the CC group as has been previously demonstrated in normally sighted individuals (Rohlf et al., 2021). Thus, the present results provide further evidence for a dissociation of cumulative crossmodal spatial recalibration (VAEc) and both immediate crossmodal recalibration (VAEi) and multisensory integration (VE), suggesting that the latter two processes originate from overlapping neural mechanisms (Park and Kayser, 2019).

The results for the VE contrast with previous reports of an impaired integration of visual and auditory speech signals in CC individuals (Putzar et al., 2007, 2010). However, we think there is a crucial difference between audio-visual speech perception and audio-visual localization: In contrast to localization, speech perception is better achieved through the auditory than through the visual (lipreading) channel. Prospective studies have shown that the McGurk effect, that is, the fusion of incongruent auditory and visual speech signals, increases between 3 and 9 years of age (Hirst et al., 2018). Younger children more likely reported the auditory input and needed a relatively more reliable visual stimulus in order to report a fused percept (Hirst et al., 2018). By contrast, the VE was stronger in children than in adults (Rohlf et al., 2020), that is, the VE declines rather than increases with age. Thus, in both cases more adult-like multisensory percepts arise across development from an increased weighting of the less reliable (less dominant) sensory input. This requires an increased weighting of vision in audio-visual speech perception, but an increased weighting of the auditory input for audio-visual localization (and hence a reduction of the visual influence). In CC individuals, the latter is likely already in place but the first can only be developed after sight restoration. Thus, the different developmental time course for audiovisual speech and spatial processes might have favored recovery of multisensory spatial integration despite the remaining and often quite severe post-surgical visual impairments typically seen in CC individuals. Our modeling results suggest that CC individuals weighted auditory and visual spatial information according to their current relative reliability similar to normally sighted individuals. A typical VE in CC individuals does, however, not necessarily mean that the VE does not require visual or crossmodal input to emerge, but rather that this visual or crossmodal input is not required during the first phase of life (Senna et al., 2021).

These results are seemingly at odds with studies in dark reared cats in which no multisensory enhancement was found in multisensory neurons of the superior colliculus (Wallace et al., 2004; Yu et al., 2019). Recently, however, multisensory gains in superior colliculus neurons have been observed when the animals were tested in light rather than in the dark (Smyre et al., 2021). We conducted our experiment in a lit room and, moreover, unlike the previously tested dark-reared cats, the CC individuals of the present study had extensive experience with audio-visual stimuli in natural environments after cataract-removal surgery, many for several years. This experience might have allowed for a recovery of the typical visual influence on auditory localization. A prerequisite for such a recovery is that unisensory visual spatial representations recover after sight restoration. In fact, a regular visual topographic organization has been demonstrated in visually deprived owls (Du Lac and Knudsen, 1991) and ferrets (King and Carlile, 1993) that were raised with binocular lid suture, and for primary visual cortex in human CC individuals (Sourav et al., 2018). Moreover, both in owls (Knudsen et al., 1991) and ferrets (King and Carlile, 1993) at least a crude alignment of auditory and visual receptive fields was observed immediately after terminating visual deprivation. It is possible that diffuse residual light perception, which exists even in the presence of bilateral dense cataracts similarly as in lid-sutured animal models (Sherman and Spear, 1982), had contributed to the preservation of the typical crossmodal connectivity in the CC individuals of the present study.

Why did recovery in CC individuals differ between multisensory integration (VE) and recalibration (VAEc)? There is multiple independent evidence suggesting that multisensory integration (VE) and crossmodal recalibration (VAEc) reflect at least partially distinct mechanisms: Event-related potential studies revealed an early versus late modulation for the VAEc and VE, respectively (Bonath et al., 2007; Bruns et al., 2011; Bruns and Röder, 2010). Prospective studies in children have recently observed that the VE emerges prior to the VAEc (Rohlf et al., 2020, 2021), thus providing developmental evidence for a dissociation of both effects. Moreover, the VAEc was found to be independent of cue reliability whereas the VE, in accord with an extensive literature (Alais and Burr, 2019), varied with cue reliability even in the youngest (5 years old) children tested (Rohlf et al., 2020, 2021). Zaidel et al. (2011) suggested that crossmodal recalibration uses a fixed-ratio weighting of the crossmodal cues which is independent of current cue reliability (but see recent results from Hong et al., 2021). Although multisensory integration aims at improving precision and, thus, should take cue reliability into account, crossmodal recalibration might aim at achieving internal consistency (i.e., accuracy) of individual cues which does not depend on reliability (Zaidel et al., 2011).

If crossmodal recalibration indeed uses a fixed-ratio weighting of cues, the question arises how this ratio is set. The result that crossmodal recalibration emerges relatively late (>7 years) in development (Rohlf et al., 2020) suggests an experience-dependence. Recent studies have provided evidence that crossmodal temporal biases with a high intra-individual stability are altered in CC individuals, suggesting that crossmodal settings are indeed acquired and stabilized during a sensitive phase of development (Chen et al., 2017; Badde et al., 2020). Similarly, the fixed-ratio weighting of sensory cues during crossmodal recalibration seems to have a high stability and consistency across humans and other primates (Zaidel et al., 2011) and, thus, might be stabilized based on experience during a sensitive period. This is supported by the high inter-individual consistency with which the visual VAEc emerged in the CC group. However, it should be considered that in the present study a significant group difference in the visual VAEc was only obtained between the CC and SC but not between the CC and DC groups.

In summary, we found multisensory integration to be indistinguishable across groups (no visual VE emerged in the CC group) whereas for cumulative crossmodal calibration we observed an expansion to recalibrating visual localization by auditory input in the CC group. We speculate that the auditory VE, which seems to emerge early in development (Rohlf et al., 2020) and is highly dependent on cue reliability both in children (Rohlf et al., 2020, 2021) and in adults (Alais and Burr, 2004), indicates a multisensory process which has to maintain a high level of plasticity throughout life to be able to instantly adjust to changes in the environment because of the need to maximize localization precision. By contrast, crossmodal recalibration is mainly important for maintaining localization accuracy. After the body has reached a certain level of maturity, expected future adaptations are relatively minor for the majority of the population (similar as for crossmodal temporal biases, Badde et al., 2020). If experience is expected to remain stable in the population after a certain developmental phase, a sensitive period is an efficient evolutionary strategy (Frankenhuis and Walasek, 2020).

### Limitations of the study

Our results demonstrate that early crossmodal experience is not necessary for the emergence of normal multisensory spatial integration (VE) and crossmodal recalibration (auditory VAEc) capabilities, but that lack of vision during early development results in the establishment of additional recalibration mechanisms (visual VAEc) not seen in sighted individuals or individuals with a later developed visual impairment. Because of the rare patient group tested in our study, our statistical analyses had to be based on a relatively small sample size. However, the typical pattern of multisensory integration and learning (auditory VE, VAEi, and VAEc) was replicated in all three groups of the present study, and the additional visual VAEc observed in the CC group was highly consistent across CC individuals. All these effects robustly emerged despite the use of a dual-task paradigm in which participants had to report both the auditory and the visual location in crossmodal trials and which might, thus, have diminished integration of the crossmodal cues compared to a joint report. Our behavioral findings can, however, not provide direct insights into the possible neural basis for the change in recalibration mechanisms resulting from a lack of early visual experience. Besides, we are not able to precisely delineate the timing of the sensitive period because we had to rely on naturally occurring cases of blindness and sight recovery in our human model. We applied stringent selection criteria for CC individuals based on medical records to make total and dense cataract at birth likely. Nevertheless, some uncertainty about the precise morphology of the cataractous lens inevitably remains in patients who presented for surgery long after birth. In addition, although the age of cataract onset is known in CC individuals (congenital), it is not clearly defined in DC individuals because of the typically gradual onset of developmental cataracts. However, because age at surgery varied considerably but results were quite consistent across CC individuals (see Figure S8), our findings point to a crucial role of the first year in human brain development in accord with neuroanatomical studies (Gilmore et al., 2020).

## STAR★Methods

### Key resources table

**Table:** 

REAGENT or RESOURCE	SOURCE	IDENTIFIER
**Deposited data**		

Raw behavioral data	This paper	https://doi.org/10.25592/uhhfdm.10191

**Software and algorithms**		

Presentation	Neurobehavioral Systems, Inc.	https://www.neurobs.com/
R version 4.0.4	R Foundation for Statistical Computing	https://www.R-project.org/
JASP version 0.14	JASP Team	https://jasp-stats.org/

### Resource availability

#### Lead contact

Further information and requests for resources should be directed to and will be fulfilled by the lead contact, Patrick Bruns (patrick.bruns@uni-hamburg.de).

#### Materials availability

This study did not generate new unique reagents.

### Experimental model and subject details

A group of 11 individuals (aged 12-41 years, *M* = 25.9, *SD* = 10.1, two female and nine male) with a history of dense bilateral congenital cataracts (CC), resulting in a transient period of blindness (according to the classification of the World Health Organization, 2019) from birth, was recruited. Their cataracts were surgically removed between the ages of 5 months and 33 years (*M* =11.7 years), with surgery taking place at or before the age of two years in four individuals, at the age of six years in one individual, and at or after the age of 14 years in the remaining six individuals (for individual participant characteristics, see Table S3). It should be noted that the exact duration of blindness does not necessarily correspond to age at surgery in CC individuals with absorbed cataracts: Two individuals from our CC group who had presented for surgery with absorbed cataracts later in life (>14 years of age) did no longer fulfil the World Health Organization (2019) criteria for blindness (but still had severe vision impairment) at the time of surgery (see Table S3). However, although the exact age of cataract absorption in these two individuals is unknown to us, cataract absorption typically does not occur before middle childhood, suggesting that they had experienced an extended period of blindness from birth. Cataract history, as well as associated factors such as presence of sensory nystagmus, absence of fundus view prior to surgery, and positive family history, were confirmed by medical records. Data of one additional CC individual who was operated only in one eye at the time of testing were not included in the analyses because of poor vision in the operated eye (logMAR visual acuity of 1.55). The remaining CC individuals had a mean logMAR visual acuity of 0.81 (range: 0.21 to 1.29).

An additional group of 10 individuals (aged 9-28 years, *M* = 14.8, *SD* = 5.2, seven female and three male) who were treated for developmental cataracts (DC) served as a control group (for individual participant characteristics, see Table S3). The DC individuals had a mean logMAR visual acuity of 0.24 (range: −0.05 to 0.63). A Welch two-sample *t* test revealed a significant difference in visual acuity between the CC and the DC group, *t*(18.78) = 4.44, *p*<.001. All CC and DC individuals reported normal hearing and had no known neurological disorders. Cataract-reversal individuals in the CC and DC groups were compared to a group of 10 normally sighted control (SC) individuals (aged 15-30 years, *M* = 19.1, *SD* = 4.8, three female and seven male) who had a typical development of all sensory systems. All 10 SC individuals had normal or corrected-to-normal vision according to their self-report. Additional visual acuity data were available for seven SC individuals who had a mean logMAR acuity of -0.17 (range: −-0.24 to −0.10). The age range of the SC group (15-30 years) largely overlapped with the age ranges of the CC and DC groups, except for the youngest participants in the DC group (minimal age of 9 years) and the oldest participants in the CC group (maximal age of 41 years). However, previous studies had shown that the experimental effects of interest, the VE and VAE, were adult-like in 8-9 year-old children (Rohlf et al., 2020) and did not differ between younger and older adults (Stawicki et al., 2019) or only between younger adults and adults aged above 60 years (Park et al., 2021).

All CC, DC, and SC individuals were recruited at the LV Prasad Eye Institute in Hyderabad, India. Participants or, for minors, their legal guardians provided written informed consent prior to taking part in the study. Expenses associated with taking part in the study, such as travel costs, were reimbursed and minors received a small gift. The study was approved by the Local Ethics Committee of the Faculty of Psychology and Human Movement at the University of Hamburg, Germany, as well as by the Institutional Review Board at the LV Prasad Eye Institute in Hyderabad, India, and was performed in accordance with the ethical standards laid down in the Declaration of Helsinki.

### Method details

#### General procedure

The experimental setup resembled those used in typical studies of the VE and VAE (Frissen et al., 2003; Jackson, 1953; Körding et al., 2007; Park and Kayser, 2019, 2021; Rohe and Noppeney, 2015; Wozny et al., 2010; Wozny and Shams, 2011; Zierul et al., 2017). Participants faced the center of four loudspeakers (Companion 2, Bose Corporation, Framingham, MA, USA) which were placed at a distance of 45 cm with eccentricities of ±5° and ±15° from the participants’ straight-ahead position (0°). The loudspeakers were covered by an acoustically transparent cloth. Four pairs of red LEDs were attached on top of the loudspeaker array, one pair at the center of each loudspeaker location. LED pairs rather than single LEDs were used to increase the saliency of the visual stimulation because we were concerned that residual unimodal visual localization impairments in CC individuals (and possibly in DC individuals) would have otherwise masked any genuine group differences in multisensory processing (Badde et al., 2020). For visual stimulation, LEDs were illuminated for 35 ms. Auditory stimuli were white noise bursts with a duration of 35 ms. Audiovisual stimuli were always presented synchronously. Participants indicated perceived stimulus locations with an array of 12 pushbuttons which was placed directly in front of the loudspeakers and which had the same width as the loudspeaker array. A schematic illustration of the experimental setup is shown in Figure 1.

The experiment consisted of two blocks of 240 trials each. Block 1 was designed to measure VE and VAEi, and Block 2 was designed to measure VAEc. Each block lasted approximately 15 min and included 40 unimodal auditory (A) trials, 40 unimodal visual (V) trials, and 160 audiovisual (AV) trials. After each trial, participants indicated the perceived location of the A and/or V stimulus by pressing the corresponding button in front of the loudspeaker/LED array. On AV trials, two responses were required, one for the A and one for the V stimulus. Half of the participants reported A first and V second throughout the experiment, and vice versa for the other half of the participants. The task was self-paced and accuracy was stressed over response speed. The next trial was presented between 900 and 1400 ms (randomly determined) after the (second) response.

#### Block 1 (VE and VAEi)

A and V trials were equally distributed across the four locations (10 trials per location each). In AV trials, each possible combination of the four A and V locations occurred 10 times, resulting in AV spatial discrepancies of ±30° (10 trials each), ±20° (20 trials each), ±10° (30 trials each), or 0° (40 trials). A, V, and AV trials were presented in a pseudorandom order, with the constraint that A and V trials were equally often preceded (disregarding any interjacent unimodal trials) by AV trials with ±20°, ±10°, and 0° spatial discrepancy (8 trials for each preceding AV discrepancy). AV trials with ±30° discrepancy were never immediately followed by a unimodal trial.

#### Block 2 (VAEc)

A and V trials were equally distributed across the four locations as in Block 1. However, in AV trials the V stimulus was always 10° to the right of the A stimulus. Thus, only three combinations of A and V locations were presented (A:-15°, V:-5°, 53 trials; A:-5°, V: 5°, 54 trials; A: 5°, V: 15°, 53 trials). A, V, and AV trials were presented in random order.

### Quantification and statistical analysis

#### VE, VAEi, and VAEc

As in previous studies using categorical localization responses (Bruns et al., 2011; Bruns and Röder, 2010; Zierul et al., 2017), individual responses were coded as 1-12 (corresponding to the 12 pushbuttons from left to right) and then averaged across the 10 unimodal trials (A and V separately) per location from Block 1. These baseline localization scores were then subtracted from localization responses in each trial of Block 1 and 2 to determine signed localization errors (negative indicates leftward and positive indicates rightward localization bias). Localization errors were converted from response units to degrees for visualization purposes.

Ventriloquism effects (VE) were calculated as difference in localization errors between AV trials with a rightward discrepancy (V to the right from A location) and AV trials with a leftward discrepancy (V to the left from A location) in Block 1. Immediate ventriloquism aftereffects (VAEi) were calculated as difference in localization errors between unimodal trials which were preceded by an AV trial with a rightward discrepancy and unimodal trials which were preceded by an AV trial with a leftward discrepancy in Block 1. Cumulative ventriloquism aftereffects (VAEc) were calculated as mean localization errors in unimodal trials of Block 2, which indicate the difference in localization responses between Block 1 (average AV discrepancy of 0°) and Block 2 (constant AV discrepancy of 10°). VE, VAEi, and VAEc were each calculated separately for A and V responses. Correlations between VE, VAEi, and VAEc effects as well as correlations between effects and logMAR visual acuity were calculated as Spearman’s rank correlations.

The size of the auditory and visual VE, VAEi, and VAEc was compared between groups by means of Welch ANOVAs and Games-Howell post-hoc comparisons. In addition, one-tailed, one-sample *t* tests were used to test whether effects were significantly larger than zero (Holm-corrected for multiple comparisons). All tests were additionally performed as Bayesian hypothesis tests using standard priors in JASP Version 0.14 (Wagenmakers et al., 2018) and Bayes Factors (BF_10_ for two-tailed tests and BF_+0_/BF_-0_ for one-tailed tests) are reported to indicate the evidential value for the null or alternative hypothesis, respectively.

#### Model descriptions

We conducted modeling analysis to probe the computational components of the multisensory integration and learning processes in CC, DC, and SC individuals. Audiovisual integration is well described by Bayesian hierarchical causal inference models (Kayser and Shams, 2015; Körding et al., 2007; Wozny and Shams, 2011). The general idea is that cue integration improves perceptual precision and accuracy only when the cues provide redundant information about the same object or event, otherwise the cues should be processed separately. Therefore, the perceptual system needs to perform causal inference to determine which cues originated from the same cause and should be integrated. The causal inference (CI) models estimate how likely the auditory and visual cues share a common cause and arbitrate between integrating and segregating the cues accordingly. Not only do these models provide normative benchmarks against which behavioral VE data can be compared, they also allow quantitative assessment of any changes associated with the VAE, which are reflected in the changes of model parameters (Wozny and Shams, 2011).

We considered three different CI models, as well as four baseline models, for the audiovisual localization responses of each participant. All the models assume that the auditory (A) and visual (V) stimuli at the locations (s_A_, s_V_) give rise to noisy internal sensations (x_A_, x_V_), and that (x_A_, x_V_) vary from trial to trial even for the same (s_A_, s_V_). The models have access only to the noisy (x_A_, x_V_) and must estimate (s_A_, s_V_). Below we describe the important components of the CI models and the baseline models.

**The Causal Inference (CI) Models.** The CI models infer the unknown causal structure (C) of the stimuli in order to estimate the true locations (s_A_, s_V_). A and V may have a common cause (C = 1) or independent causes (C = 2). If A and V are caused by the same object (C = 1), then the optimal approach to estimate the one true location (s = s_A_ = s_V_) is to integrate the model’s A and V location estimates (${\hat{S}}_{A\;},\;{\hat{S}}_{V}$) according to the relative reliabilities of the A and V sensory cues. This approach is often referred to as optimal integration (Alais and Burr, 2004; Rohde et al., 2016) or forced fusion (De Winkel et al., 2015). If A and V are caused by independent objects (C = 2), then the true A and V locations should be separately estimated, based solely on each unimodal sensory cue. The CI models are probabilistic models that represent information uncertainty in terms of probabilistic distributions (Li et al., 2020). Each CI model takes the perspective of a Bayesian ideal observer (Geisler, 2011), which combines sensory evidence and prior belief to calculate the probability of the underlying causal structure C, according to the Bayes’ rule:

(Equation 1) $$p\left(C|x_{A},x_{V}\right)=\frac{p\left(x_{A},x_{V}|C\right)p\left(C\right)}{p\left(x_{A},x_{V}\right)}$$

Here, C could be 1 (common cause) or 2 (independent causes), and $p\left(C|x_{A},x_{V}\right)$ is the posterior probability (“posterior”) of C given the sensory data (x_A_, x_V_). The prior probability (“prior”) for the causal structure is denoted as p(C). Specifically, the prior for a common cause, p(C = 1), is known as the *causal prior* (Körding et al., 2007) and hereafter denoted as Pc for short. Pc represents the prior belief about how likely the cues share a common cause. Because the models only consider two possible causal structures, the prior for independent causes, p(C = 2), simply equals 1 – Pc. The likelihood function (“likelihood”), $p\left(x_{A},x_{V}|C\right)$, calculates the joint probability of the sensory data (x_A_, x_V_) given a certain causal structure C. The likelihood quantifies the probabilistic mapping between the physical environment and the internal sensory representations.

For a common cause (C = 1), the posterior probability in Equation (1) is

(Equation 2) $$p\left(C=1|x_{A},x_{V}\right)=\frac{p\left(x_{A},x_{V}|C=1\right)P_{C}}{p\left(x_{A},x_{V}|C=1\right)P_{C}+p\left(x_{A},x_{V}|C=2\right)\left(1-P_{C}\right)}$$

Like the priors, the posteriors sum to one; therefore, the posterior for C = 2, $p\left(C=2|x_{A},x_{V}\right)$, simply equals 1 - p$\left(C=1|x_{A},x_{V}\right)$. The joint likelihood of getting the sensory data (x_A_, x_V_) under C = 1 is calculated by integration:

(Equation 3) $$p\left(x_{A},x_{V}|C=1\right)=\int p\left(x_{A},x_{V}|s\right)p\left(s\right)ds=\int p\left(x_{A}|s\right)p\left(x_{V}|s\right)p\left(s\right)ds$$

where s = s_A_ = s_V_. The *location prior*, p(s), is the prior probability distribution over the possible stimulus locations; it represents the expectation of where the stimulus is likely to occur. Given a stimulus at location s, $p\left(x_{A}|s\right)$ and $p\left(x_{V}|s\right)$ are the unimodal likelihoods of sensing x_A_ and x_V_, respectively. Assuming Gaussian distributions for p(s), $p\left(x_{A}|s\right)$, and $p\left(x_{V}|s\right)$, Equation (3) can be analytically solved as

(Equation 4) $$p\left(x_{A},x_{V}|C=1\right)=\frac{1}{2\pi \sqrt{\sigma_{A}^{2}\sigma_{V}^{2}+\sigma_{A}^{2}\sigma_{P}^{2}+\sigma_{V}^{2}\sigma_{P}^{2}}}exp\left[-\frac{1}{2}\frac{{\left(x_{V}-x_{A}\right)}^{2}\sigma_{P}^{2}+{\left(x_{A}-\mu_{P}\right)}^{2}\sigma_{V}^{2}+{\left(x_{V}-\mu_{P}\right)}^{2}\sigma_{A}^{2}}{\sigma_{A}^{2}\sigma_{V}^{2}+\sigma_{A}^{2}\sigma_{P}^{2}+\sigma_{V}^{2}\sigma_{P}^{2}}\right]$$

Here, $\mu_{P}$ and σ_P_ are the mean and standard deviation of the Gaussian location prior p(s), and σ_A_ and σ_V_ are the standard deviations of the Gaussian noises that have corrupted the unisensory A and V signals, respectively.

By contrast, if the cues have independent causes (C = 2), then the joint likelihood of observing the sensory data (x_A_, x_V_) is calculated by

(Equation 5) $$p\left(x_{A},x_{V}|C=2\right)=\iint p\left(x_{A},x_{V}|s_{A},s_{V}\right)p\left(s_{A},s_{V}\right)ds_{A}ds_{V}=\left(\int p\left(x_{A}|s_{A}\right)p\left(s_{A}\right)ds_{A}\right)\left(\int p\left(x_{V}|s_{V}\right)p\left(s_{V}\right)ds_{V}\right)$$

where s_A_ and s_V_ independently give rise to x_A_ and x_V_. Again, assuming Gaussian distributions for $p\left(s_{A}\right)$, $\;p\left(s_{V}\right)$, $p\left(x_{A}|s_{A}\right)$, and $p\left(x_{V}|s_{V}\right)$, Equation 5 has the analytical solution

(Equation 6) $$p\left(x_{A},x_{V}|C=2\right)=\frac{1}{2\pi \sqrt{\left(\sigma_{A}^{2}+\sigma_{P}^{2}\right)\left(\sigma_{V}^{2}+\sigma_{P}^{2}\right)}}exp\left[-\frac{1}{2}\left(\frac{{\left(x_{A}-\mu_{P}\right)}^{2}}{\sigma_{A}^{2}+\sigma_{P}^{2}}+\frac{{\left(x_{V}-\mu_{P}\right)}^{2}}{\sigma_{V}^{2}+\sigma_{P}^{2}}\right)\right]$$

A common method to obtain optimal estimation is to minimize the mean squared error as the cost function (Körding et al., 2007):

(Equation 7) $$Cost={\left({\hat{S}}_{A\;}-S_{A}\right)}^{2}+\;{\left({\hat{S}}_{V\;}-S_{V}\right)}^{2}$$

Minimizing this cost function under C = 1 will give the analytical solution

(Equation 8) $${\hat{s}}_{A,\;C=1}={\hat{s}}_{V,\;C=1}=\frac{\frac{x_{A}}{\sigma_{A}^{2}}+\frac{x_{V}}{\sigma_{V}^{2}}+\frac{\mu_{P}}{\sigma_{P}^{2}}}{\frac{1}{\sigma_{A}^{2}}+\frac{1}{\sigma_{V}^{2}}+\frac{1}{\sigma_{P}^{2}}}$$

This is the optimal estimate if C = 1. Equation (8) is a more general form of the optimal integration model (Alais and Burr, 2004; Ernst and Banks, 2002). If the models do *not* account for the location prior (which is equivalent to using a uniform location prior: $\mu_{P}$ = 0, $\sigma_{P}\to \infty$), then Equation (8) reduces to the optimal integration model, which weights the sensory data by their relative reliabilities, and the reliability is represented by the inverse variance 1/$\sigma_{A}^{2}$ or 1/$\sigma_{V}^{2}$.

Under C = 2, minimizing the cost function in Equation (7) will give the analytical solutions for the A and V estimates separately as:

(Equation 9a) $${\hat{s}}_{A,\;C=2}=\frac{\frac{x_{A}}{\sigma_{A}^{2}}+\frac{\mu_{P}}{\sigma_{P}^{2}}}{\frac{1}{\sigma_{A}^{2}}+\frac{1}{\sigma_{P}^{2}}}$$

(Equation 9b) $${\hat{s}}_{V,\;C=2}=\frac{\frac{x_{V}}{\sigma_{V}^{2}}+\frac{\mu_{P}}{\sigma_{P}^{2}}}{\frac{1}{\sigma_{V}^{2}}+\frac{1}{\sigma_{P}^{2}}}$$

These are the optimal estimates if C = 2, which are equivalent to estimating the A and V locations independently, based solely on the respective unisensory data.

The three CI models we considered are identical in applying Equations (1, 2, 3, 4, 5, 6, 7, 8, 9a, and 9b) but differ in how they combine the estimates obtained under C = 1 (integration) and C = 2 (segregation) to generate the responses for A and V localization. The optimal strategy to combine the estimates is model averaging (MA), because it results in the minimal mean expected squared error (Körding et al., 2007). The CI model with the MA strategy (CI-MA) calculates the weighted averages of the estimates under C = 1 and C = 2; the corresponding weights are the posterior probabilities of C = 1 and C = 2:

(Equation 10a) $${\hat{S}}_{A}=p\left(C=1|X_{A},\;X_{V}\right){\hat{S}}_{A,\;C=1}+\left(1-\text{p}\left(C=1|X_{A},\;X_{V}\right)\right)\;{\hat{S}}_{A,\;C=2}$$

(Equation 10b) $${\hat{S}}_{V}=p\left(C=1|X_{A},\;X_{V}\right){\hat{S}}_{V,\;C=1}+\left(1-\text{p}\left(C=1|X_{A},\;X_{V}\right)\right)\;{\hat{S}}_{V,\;C=2}$$

In addition to the MA strategy, behavioral studies on causal inference have shown that humans and non-human primates sometimes apply two heuristic alternatives for audiovisual localization (Mohl et al., 2020; Wozny et al., 2010): probability matching (PM) and model selection (MS). The CI model with the PM strategy (CI-PM) chooses between the estimates under C = 1 and C = 2 with the posterior probabilities of C = 1 and C = 2:

(Equation 11a) $${\hat{S}}_{A}=\left\{ \begin{array}{l} {\hat{S}}_{A,\;C=1}\;if\;p\left(C=1|X_{A},\;X_{V}\right)>\xi \\ {\hat{S}}_{A,\;C=2}\;if\;p\left(C=1|X_{A},\;X_{V}\right)\leq \xi \end{array}\right.$$

(Equation 11b) $${\hat{S}}_{V}=\left\{ \begin{array}{l} {\hat{S}}_{V,\;C=1}\;if\;p\left(C=1|X_{A},\;X_{V}\right)>\xi \\ {\hat{S}}_{V,\;C=2}\;if\;p\left(C=1|X_{A},\;X_{V}\right)\leq \xi \end{array}\right.$$

where ξ is sampled from a uniform distribution [0,1] on each trial. For example, if the posterior probability of C = 1 is 0.6 on a trial, then 60% of the time the CI-PM model will choose ${\hat{S}}_{A,C=1}$ as its final estimate for the A location, but 40% of the time it will choose ${\hat{S}}_{A,\;C=2}$ as its final estimate for the A location (Wozny et al., 2010). The CI model with the MS strategy (CI-MS) compares the posterior probabilities of C = 1 and C = 2 and simply uses the estimates from the more probable causal structure, that is, the causal structure whose posterior probability is >0.5:

(Equation 12a) $${\hat{S}}_{A}=\left\{ \begin{array}{l} {\hat{S}}_{A,\;C=1}\;if\;p\left(C=1|X_{A},\;X_{V}\right)>.5\\ {\hat{S}}_{A,\;C=2}\;if\;p\left(C=1|X_{A},\;X_{V}\right)\leq .5 \end{array}\right.$$

(Equation 12b) $${\hat{S}}_{V}=\left\{ \begin{array}{l} {\hat{S}}_{V,\;C=1}\;if\;p\left(C=1|X_{A},\;X_{V}\right)>.5\\ {\hat{S}}_{V,\;C=2}\;if\;p\left(C=1|X_{A},\;X_{V}\right)\leq .5 \end{array}\right.$$

For example, if the posterior probability of C = 1 is 0.6 on a trial, then the CI-MS model will choose ${\hat{S}}_{A,C=1}$ as its final estimate for the A location (Wozny et al., 2010).

**Baseline Models.** In addition to the three causal inference models (CI-MA, CI-PM, and CI-MS), we considered four baseline models. The baseline models do not perform causal inference; instead, they assume a fixed causal structure and always either fuse or segregate the A and V cues to estimate the stimulus locations.

Two of the baseline models take into account the location prior: (1) A forced fusion (FF) model with a Gaussian location prior. This model always integrates A and V sensory data based on their reliabilities according to Equation (8). The FF model assumes a common cause and is equivalent to the C = 1 branch of a CI model. (2) A segregation (SG) model with a Gaussian location prior. This model completely segregates A and V sensory data without any integration or inter-modal influence. The A and V locations are estimated independently, based solely on the information within each modality according to Equations (9a) and (9b). The SG model assumes independent causes and is equivalent to the C = 2 branch of a CI model.

To check whether the prior parameters were necessary for modeling our data, we additionally tested the simplified versions of the above two baseline models without the location prior: (3) A forced fusion model with no priors (FFnp), and (4) a segregation model with no priors (SGnp). Mathematically, having no priors is equivalent to assuming a flat location prior ($\mu_{P}$= 0, $\sigma_{P}\to \infty$), which means these models have no expectations for the stimulus location, and a fixed causal prior (Pc = 1 for FFnp, Pc = 0 for SGnp). The FFnp model is identical to the optimal integration model (Alais and Burr, 2004; Ernst and Banks, 2002) and applies a simplified version of Equation (8) without the prior parameters:

(Equation 13) $${\hat{s}}_{A,\;C=1}={\hat{s}}_{V,\;C=1}=\frac{\frac{x_{A}}{\sigma_{A}^{2}}+\frac{x_{V}}{\sigma_{V}^{2}}}{\frac{1}{\sigma_{A}^{2}}+\frac{1}{\sigma_{V}^{2}}}$$

This equation is the same as the reliability-based weighted-average formula of the optimal integration model. Similarly, the SGnp model applies a simplified version of Equations (9a) and (9b) without the prior parameters:

(Equation 14) $${\hat{s}}_{A,\;C=2}=x_{A};\;{\hat{s}}_{V,\;C=2}=x_{V}$$

#### Model Parameters and Simulations

**Causal inference models.** Each CI model has seven parameters: ${\text{Δ}}_{A}$, $\sigma_{A}$– the bias and the standard deviation (SD) of the auditory Gaussian likelihood; ${\text{Δ}}_{V}$, $\sigma_{V}$– the bias and the SD of the visual Gaussian likelihood; $\mu_{P},$$\sigma_{P}$– the mean and the SD of the Gaussian location prior for stimuli of either modality; Pc – the causal prior, that is, the prior for a common cause.

Typical CI models assume unbiased likelihoods and priors (e.g., Körding et al., 2007; Wozny et al., 2010); that is, they assume that the likelihoods center at the true stimulus locations (${\text{Δ}}_{A}$= 0, ${\text{Δ}}_{V}$= 0) and that the location prior centers at 0° azimuth angle ($\mu_{P}$= 0). Here we incorporate non-zero bias terms to probe whether the VAEc is associated with changes in the likelihood or prior biases – in other words, whether a shift in the likelihood or location prior distribution is a possible computational mechanism underlying the VAEc (for detail, see Wozny and Shams, 2011).

For each combination of the seven parameters and each combination of true audiovisual (AV) stimulus locations (s_A_, s_V_), each CI model performed 10,000 Monte Carlo simulated AV localization trials. The noisy internal sensory representations x_A_ and x_V_ were generated according to the Gaussian likelihood functions $p\left(x_{A}|s\right)$ and $p\left(x_{V}|s\right)$: On each simulated trial, x_A_ and x_V_ were independently sampled from two Gaussian distributions representing the independent Gaussian noises that corrupted the A and V signals. The Gaussian distributions centered at the true stimulus locations s_A_ and s_V_ plus the likelihood biases ${\text{Δ}}_{A}$ and ${\text{Δ}}_{V}$, respectively, with $\sigma_{A}$ and $\sigma_{V}$determining the strengths of the Gaussian noises: $x_{A}\;∼\;N\left(s_{A}+{\text{Δ}}_{A},\;\sigma_{A}\right)$ and $x_{V}\;∼\;N\left(s_{V}+{\text{Δ}}_{V},\;\sigma_{V}\right)$. Additionally, the model accounted for a prior bias for the stimulus location, represented by a Gaussian distribution centered at $\mu_{P}$, and the strength of this location prior bias was determined by $\sigma_{P}$: $p\left(s\right)=\;N(\mu_{P},\;\sigma_{P}^{}$). The model combined the likelihoods, the location prior, and the causal prior to calculate the posterior posteriors for C = 1 and C = 2, as well as estimating the A and V locations under each causal structure. Up to this step, the three CI models would produce the same estimates given a certain set of parameter values. The CI models differed in the next step: Each CI model implemented its decision strategy (MA, PM, or MS) to use these estimates to generate the responses for the A and V locations on that AV trial. The resulting 10,000 simulated A responses and 10,000 simulated V responses were then each binned into a histogram of 12 bins, corresponding to the 12 discrete button-response positions. The trial counts in the bins were then normalized into relative proportions of counts. The final output of each CI model were two histograms of proportions of counts, one for A localization and one for V localization. These were the model’s predicted AV response distributions (in proportions of counts) for a pair of AV stimulus locations (s_A_, s_V_), given a set of parameter values.

**Baseline Models.** The two baseline models with a Gaussian location prior – the forced fusion (FF) model and the segregation (SG) model – have the same parameters as the CI models except that the causal prior (Pc) is fixed instead of being a free parameter. The FF model assumes a common cause, so Pc = 1. The SG model assumes independent causes, so Pc = 0. The simulation processes of the FF model and the SG model are identical to the C = 1 and C = 2 branches, respectively, of the CI models.

The other two baseline models – forced fusion with no priors (FFnp) and segregation with no priors (SGnp) – do not have any location prior parameters. For the purpose of calculating average parameter values, the causal prior is assigned to be Pc = 1 for FFnp and Pc = 0 for SGnp. In other words, FFnp and SGnp each has only four parameters: ${\text{Δ}}_{A}$, $\sigma_{A}$ (mean bias and SD of the A likelihood) and ${\text{Δ}}_{V}$, $\sigma_{V}$ (mean bias and SD of the V likelihood).

Like the CI models, each baseline model performed 10,000 Monte Carlo simulated AV localization trials and generated model-predicted AV response distributions (in proportions of counts). For the two forced fusion models (FF and FFnp), the predicted A and V response distributions were identical, because the models assumed that A and V shared the same location on each trial. For the two segregation models (SG and SGnp), the predicted A and V response distributions were different.

#### Model fitting and comparison

**Step 1.** The unimodal A and V Gaussian likelihood biases and SDs (${\text{Δ}}_{A}$, $\sigma_{A}$, ${\text{Δ}}_{V}$, $\sigma_{V}$) were estimated based on the unimodal A and V trials of Blocks 1 and 2 separately for each participant (Zierul et al., 2019). To fit the unimodal A and V localization data, we applied the “fitdist” function from the R-package “fitdistrplus” (package version 1.1-3, R version 4.0.4), using maximum likelihood estimation (MLE) and discretizing the Gaussian distributions to match the discrete experimental data. For each participant, we obtained a set of best-fitted (${\text{Δ}}_{A}$, $\sigma_{A}$, ${\text{Δ}}_{V}$, $\sigma_{V}$) for Block 1 and another set for Block 2.

**Step 2.** We fitted and compared three CI models and four baseline models to each participant’s AV trials in Block 1, to find the individual’s best-fitting model and corresponding prior parameters (e.g., $\mu_{P},$$\sigma_{P}$, and Pc for any CI model). This step identified the possible computational components that best described the observed multisensory integration patterns (as indicated by the VE). For a given participant, all seven models used the same individually fitted unimodal parameters (${\text{Δ}}_{A}$, $\sigma_{A}$, ${\text{Δ}}_{V}$, $\sigma_{V}$) for Block 1 from Step 1. Unlike the simple Gaussian likelihoods in Step 1, the model-predicted responses in this step were complex distributions, hence we customized the fitting procedure. Again, we used the MLE method to find the model and parameters that best explained the data. For the discrete button-response outcomes, the likelihood function to be maximized was the multinomial log-likelihood (Cao et al., 2019; Körding et al., 2007):

(Equation 15) $$LL=\sum_{i}^{k}n_{i}ln\left(p_{i}\right)+lnC$$

LL denotes the multinomial log-likelihood, k = 12 is the number of button-response positions, n_i_ denotes the observed counts of experimental trials at button-response position i, p_i_ denotes the model-predicted response probability at button-response position i, and C is the multinomial coefficient determined by the observed counts. lnC is a constant for all models and parameter sets, so maximizing LL is equivalent to maximizing $\sum_{i}^{k}n_{i}ln\left(p_{i}\right)$. For each set of prior parameters of each prior-including model (i.e., the three CI models, the FF model, and the SG model), and for each pair of A and V stimulus locations (s_A_, s_V_), 10,000 simulated AV trials were run to generate 10,000 A responses and 10,000 V responses in relative proportions of counts at the button-response positions (see “Model Parameters and Simulations” above); these model-predicted proportions were entered into Equation (15) to approximate the response probabilities p_i_ and calculate the log-likelihood for (s_A_, s_V_). The log-likelihoods for all pairs of (s_A_, s_V_) were then summed to get the overall log-likelihood. The best-fitting model and prior parameters were the ones that maximized this overall log-likelihood, or equivalently, minimized its negative value (Cao et al., 2019; Körding et al., 2007). To optimize the fitting procedure, we applied the “mle” function from the R-package “stats4” (version 4.0.4) and implemented a bounded search method (L-BFGS-B) to minimize the negative log-likelihood (Byrd et al., 1995). The search was initialized using the best starting prior parameter values obtained from a uniform grid search (Mohl et al., 2020), which divided each free prior parameter space into 10 even portions (i.e., 11 values). The grid yielded 11^3^ = 1,331 initial prior parameter sets for each CI model, and 11^2^ = 121 initial prior parameter sets for the FF and the SG models.

To compare the model performances, we calculated the Bayesian Information Criterion (BIC) for each model: $BIC=-2\left(\hat{LL}\right)+k*\text{ln}\left(N\right)$, where $\left(\hat{LL}\right)$ denotes the maximum log-likelihood, k is the number of parameters, and N is the total number of data points (Neath and Cavanaugh, 2012). The BIC awards models that fit the data well but penalizes models with more parameters (i.e., more complex). For a given dataset, the best model is the one with the lowest BIC. We thus identified the best model for each participant. All participants were best-fitted by models with priors, that is, no participant was best-fitted by FFnp or SGnp.

**Step 3.** To probe whether crossmodal learning (as indicated by VAEc) was associated with changes in any likelihood or prior parameters, we conducted modeling analysis on each participant’s Block 2 data and compared the results with those from Block 1. The A and V likelihood parameters (${\text{Δ}}_{A}$, $\sigma_{A}$, ${\text{Δ}}_{V}$, $\sigma_{V}$) for Block 2 were already fitted individually in Step 1. We used these best-fitting likelihood parameters from Step 1, as well as the best model determined in Step 2, to fit each participant’s Block 2 AV trials and obtain the best-fitting prior parameters for Block 2. We assumed that the individual’s best model did not change from Block 1 to Block 2, but that the model parameters could change (Wozny and Shams, 2011). The model fitting procedure was identical to that in Step 2. There was no model comparison in this step because the same best models from Step 2 were applied.

## Data Availability

• Raw behavioral data have been deposited in the research data repository of the University of Hamburg and are publicly available as of the date of publication. The DOI is listed in the key resources table. De-identified subject characteristics are provided in Table S3. • This paper does not report original code. • Any additional information required to reanalyze the data reported in this paper is available from the lead contact upon request.
