# Supplementary material for: Audiovisual spatial recalibration but not integration is shaped by early sensory experience
Source: iScience. 2022 May 23;25(6):104439. doi: 10.1016/j.isci.2022.104439 (PMC9301879; doi:10.1016/j.isci.2022.104439)
Supplement: Document S1. Figures S1–S8 and Tables S1–S3 [file mmc1.pdf]

iScience, Volume *25*

Supplemental information

Audiovisual spatial recalibration but not integration is shaped by early sensory experience

Patrick Bruns, Lux Li, Maria J.S. Guerreiro, Idris Shareef, Siddhart S. Rajendran, Kabilan Pitchaimuthu, Ramesh Kekunnaya, and Brigitte Röder

	CI-MA	CI-PM	CI-MS	FF	SG	FFnp	SGnp
**CC**	1	8	2	0	0	0	0
**DC**	0	3	5	0	2	0	0
**SC**	0	6	0	1	3	0	0
**Total**	1	17	7	1	5	0	0

Table S1. Number of participants best fitted by each model. Related to Table 1. CI-MA: causal inference with model averaging. CI-PM: causal inference with probability matching. CI-MS: causal inference with model selection. FF: forced fusion with location prior. SG: segregation with location prior. FFnp: forced fusion with no priors (i.e., the optimal integration model). SGnp: segregation with no priors.

		Auditory Likelihood		Visual Likelihood			Prior	
		Δ_A (deg)	σ_A (deg)	Δ_V (deg)	σ_V (deg)	μ_P (deg)	σ_P (deg)	Pc
**CC (n=10)**	Block 1	1.11±0.46	6.09±0.72	0.22±0.22	2.90±0.31	2.45±3.58	50.09±10.19	0.55±0.10
	Block 2	3.06±0.51	6.10±0.75	-0.36±0.22	3.17±0.55	0.56±4.18	32.71±8.17	0.45±0.11
	Block (2 – 1)	**1.95±0.53***	0.01±0.41	**-0.58±0.14***	0.27±0.46	-1.90±6.19	-17.38±8.59	-0.10±0.12
**DC (n=11)**	Block 1	0.98±0.58	7.16±0.91	0.21±0.61	6.19±1.25	1.83±4.43	73.22±6.14	0.27±0.06
	Block 2	2.09±0.43	6.77±0.88	0.22±0.23	4.45±1.03	2.65±4.59	42.69±9.89	0.43±0.11
	Block (2 – 1)	1.11±0.85	-0.39±0.69	-0.08±0.58	-1.74±0.70	0.82±5.80	**-30.53±9.28***	0.16±0.06
**SC (n=10)**	Block 1	0.46±0.87	6.42±0.99	0.01±0.20	3.55±0.60	-0.62±3.70	61.46±11.13	0.48±0.13
	Block 2	2.77±0.44	6.00±0.80	0.07±0.26	2.48±0.24	8.44±3.25	47.98±10.49	0.38±0.12
	Block (2 – 1)	**2.32±0.60***	-0.42±0.36	0.07±0.19	-1.07±0.47	9.06±4.23	-13.48±6.74	-0.10±0.07

Table S2. **Mean ± SEM model parameter estimates for each group. Related to Table 1.** Table based on individual participants' best-fitting models and parameters of Block 1 and Block 2. The VAEc corresponds to a change in the likelihood bias parameter (Δ_A, Δ_V) of our computational models from Block 1 (in which AV trials had a mean spatial discrepancy of 0°) to Block 2 (in which AV trials featured a constant spatial discrepancy of 10°). We additionally tested for changes in the SD of the likelihood (σ_A, σ_V), the mean and SD of the location prior (μ_P, σ_P), and/or the causal prior (Pc) by comparing the changes in the parameter values (Block 2 – Block 1) with zero, using one-sample two-tailed *t* tests (asterisks and boldface indicate significant changes from Block 1 to Block 2). Additionally, the changes from Block 1 and Block 2 in each of these five parameters (σ_A, σ_V, μ_P, σ_P, Pc) were compared among groups using Welch ANOVAs and Games-Howell post-hoc comparisons. There were no significant group differences for changes in the SD of the auditory (σ_A) [$F(2,17.63) = 0.32$, $p = .731$; $BF_{10} = 0.24$] or visual (σ_V) likelihood [$F(2,17.99) = 3.43$, $p = .055$; $BF_{10} = 0.54$] as well as for the location prior mean (μ_P) [$F(2,18.23) = 1.27$, $p = .305$; $BF_{10} = 0.41$] or SD (σ_P) [$F(2,18.32) = 1.08$, $p = .359$; $BF_{10} = 0.43$]. However, a significant group difference was observed for the causal prior (Pc) [$F(2,17.91) = 4.97$, $p = .019$; $BF_{10} = 1.23$] which was mainly due to a significant difference between the DC and SC groups [$p = .022$] whereas the CC group did not differ significantly from either group [both $p \geq .158$]. However, the change of the causal prior (Pc) from Block 1 to Block 2 was not significant in any of the three groups. *$p < .05$ (Holm-corrected over seven parameters) denotes significant changes from Block 1 to Block 2 within each group.

Subject	Age at test (years)	Gender	Age at surgery (months)	VA at test (logMAR)	Pre-op VA in better eye	Nys	Strab	Other ophthalmic disorders
CC-1	26	Male	5	0.75	Unknown	Yes	ET	Irregular iris and pupil (BE)
CC-2	37	Male	24	0.21	Unknown	Yes	ET	Retinal lattice/holes (BE)
CC-3	33	Male	72	1.29	Unknown	Yes	No	None
CC-4*	36	Female	396	1.04	20/600	Yes	XT	None
CC-5	31	Male	168	1.04	Unknown	Yes	ET	None
CC-6*	16	Male	197	0.83	20/320	Yes	No	None
CC-7*	41	Male	248	0.97	20/300	Yes	No	None
CC-8*	16	Male	186	1.15	20/600	Yes	XT	None
CC-9	19	Male	213	0.60	CF at 0.5 m	Yes	XT	Secondary glaucoma (LE)
CC-10	18	Female	25	0.51	FFL at 1 m	Yes	ET	Microcornea (BE)
CC-11	12	Male	6	0.54	PL	Yes	ET	Microcornea (BE)
DC-1	14	Female	130	0.37	20/126	No	XT	None
DC-2	9	Female	76	0.63	20/80p	Yes	ET	Amblyopia (LE)
DC-3	16	Female	196	0.58	20/30p	No	No	None
DC-4	13	Female	158	0.24	20/200	No	No	None
DC-5	15	Female	91	-0.13	20/60p	No	No	None
DC-6#	28	Male	-	0.34	-	No	No	None
DC-7	11	Female	123	0.35	20/60	No	(X)T	None
DC-8	16	Male	154	-0.05	20/60	No	No	None
DC-9	15	Female	37	0.15	20/760	No	(X)T+RHT	None
DC-10	11	Male	39	-0.05	Unknown	No	No	None

Table S3. Participant characteristics of CC and DC individuals. Related to STAR Methods. CC = congenital cataract; DC = developmental cataract; VA = visual acuity; logMAR = logarithm of the Minimum Angle of Resolution; CF = counting fingers; FFL = fixate and follow light; PL = perception of light; Nys = nystagmus; Strab = strabismus; ET = esotropia; XT = exotropia; (X)T = intermittent exotropia; RHT = right hypertropia; BE = both eyes; LE = left eye; * participant with absorbed cataract; # participant did not undergo surgery.

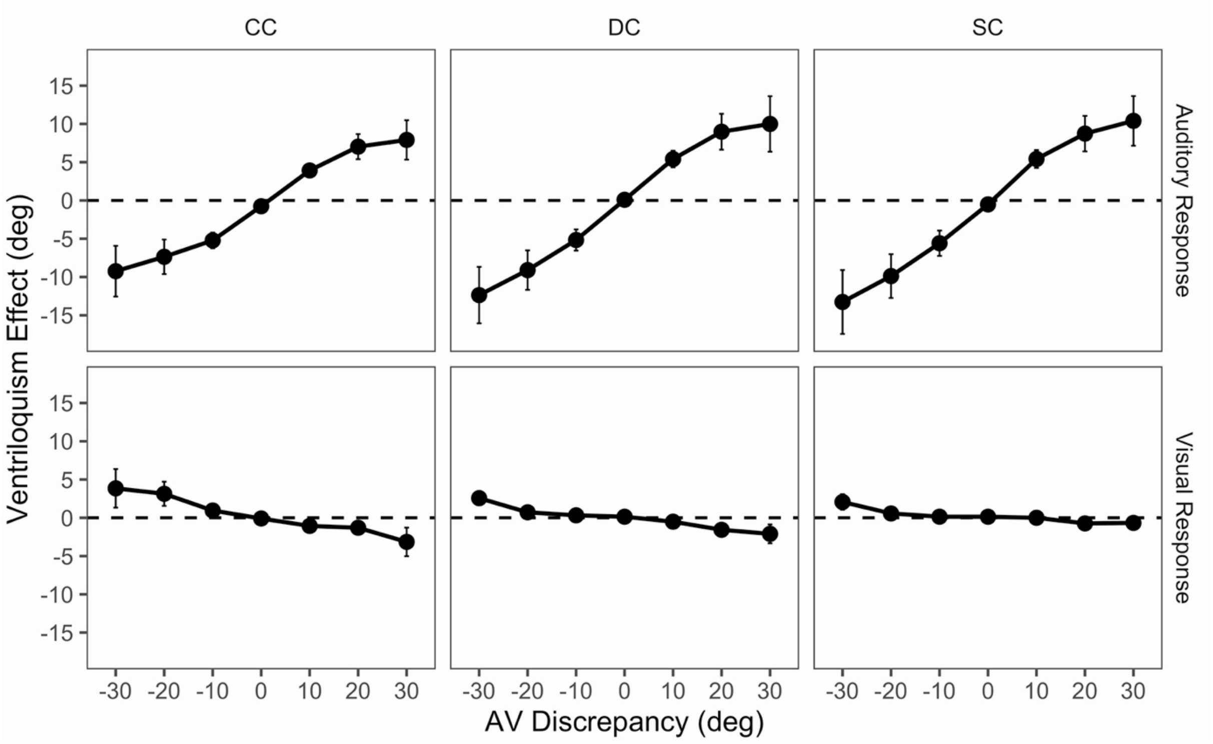

Figure S1. Mean localization responses (in degrees) in AV trials (relative to unimodal A and V trials) as a function of AV spatial discrepancy in CC, DC, and SC individuals. Related to Figure 2. Positive AV discrepancies indicate AV trials with a rightward spatial discrepancy (V to the right of A) and negative AV discrepancies indicate AV trials with a leftward discrepancy (V to the left of A). Positive *y*-axis values indicate rightward localization biases and negative values indicate leftward localization biases. Error bars indicate *SEM*s.

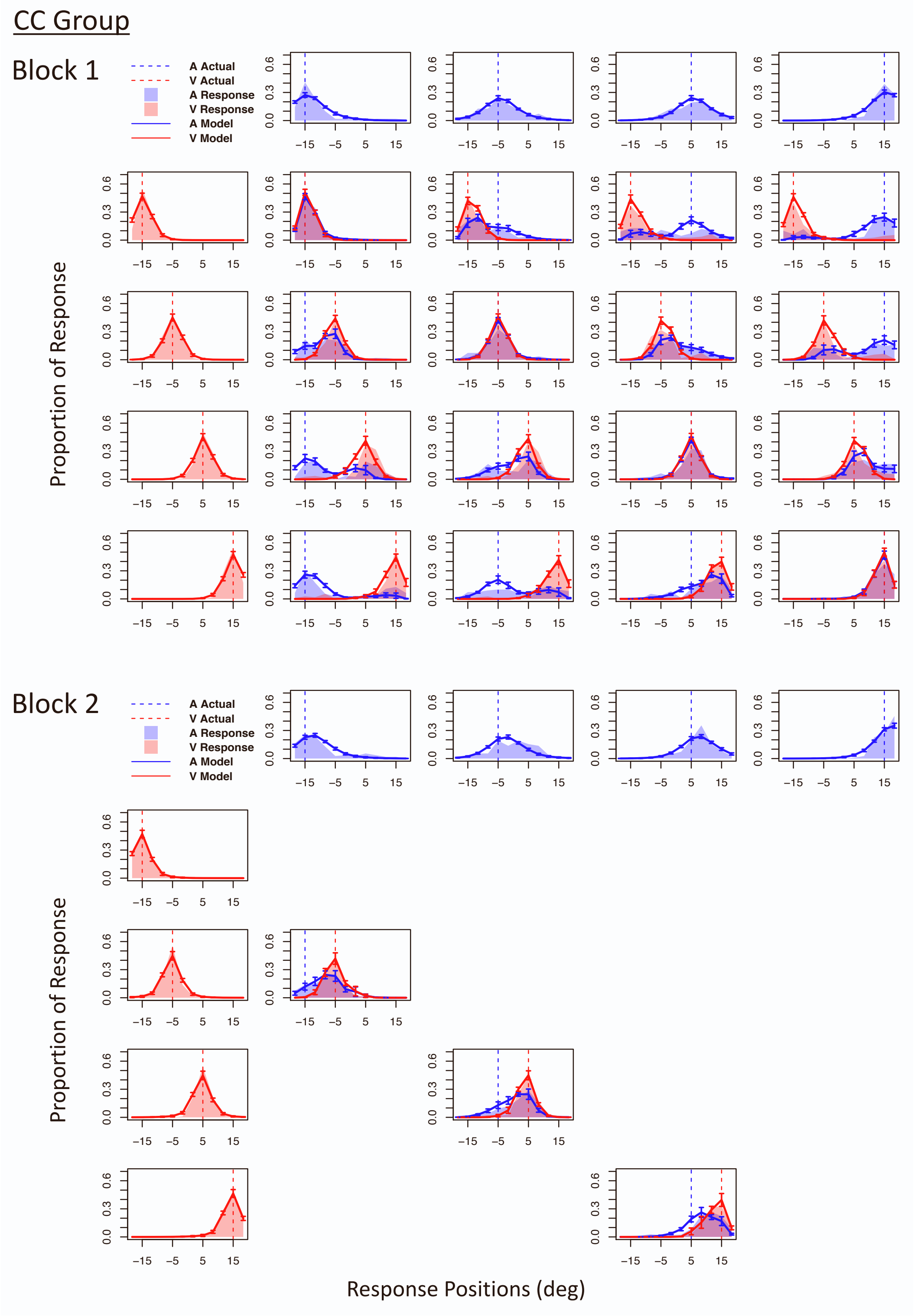

Figure S2. Auditory (A) and visual (V) localization response distributions and model best fits of Block 1 (VE/VAEi) and Block 2 (VAEc) for group CC. Related to Table 1, Figure 2, and Figure 5. Blue: A localization. Red: V localization. Vertical dotted lines indicate the actual stimulus locations. Shaded areas illustrate the proportion of responses at each response button position. Solid lines are the individually fitted model predictions (mean ± SEM), simulated using each participant's best-fitting model and parameters and then averaged within the group. In each sub-figure, the top row shows the unimodal A trials at four A locations, the left column shows the unimodal V trials at four V locations, and the remaining panels show the bimodal audiovisual (AV) trials with both A and V localization responses. In Block 2 (bottom), there are only three bimodal panels because the AV discrepancy was constant at +10°, yielding only three possible (A, V) location combinations: (-15°, -5°), (-5°, 5°), and (5°, 15°).

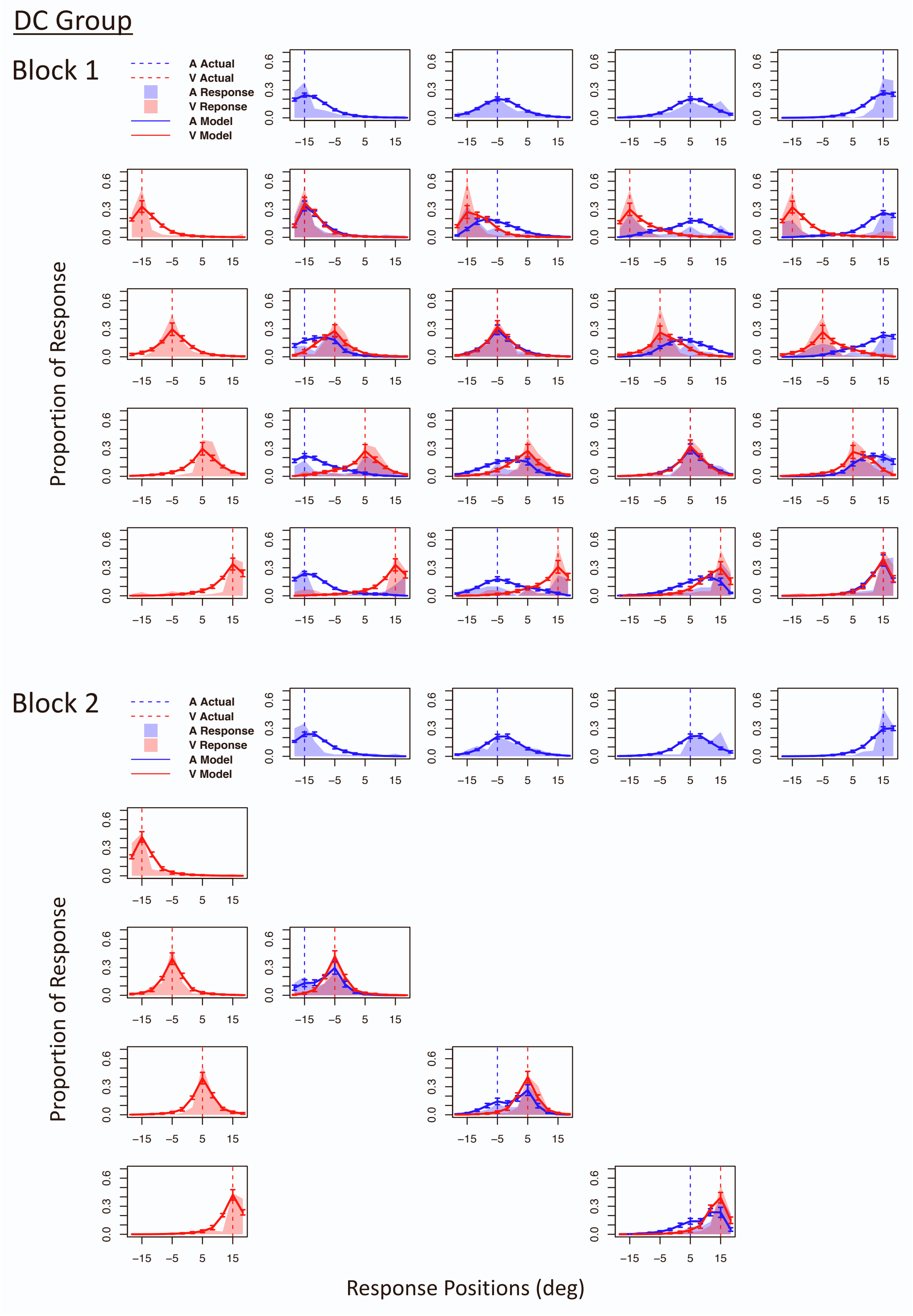

Figure S3. Auditory (A) and visual (V) localization response distributions and model best fits of Block 1 (VE/VAEi) and Block 2 (VAEc) for group DC. Related to Table 1, Figure 2, and Figure 5. Blue: A localization. Red: V localization. Vertical dotted lines indicate the actual stimulus locations. Shaded areas illustrate the proportion of responses at each response button position. Solid lines are the individually fitted model predictions (mean ± SEM), simulated using each participant's best-fitting model and parameters and then averaged within the group. In each sub-figure, the top row shows the unimodal A trials at four A locations, the left column shows the unimodal V trials at four V locations, and the remaining panels show the bimodal audiovisual (AV) trials with both A and V localization responses. In Block 2 (bottom), there are only three bimodal panels because the AV discrepancy was constant at +10°, yielding only three possible (A, V) location combinations: (-15°, -5°), (-5°, 5°), and (5°, 15°).

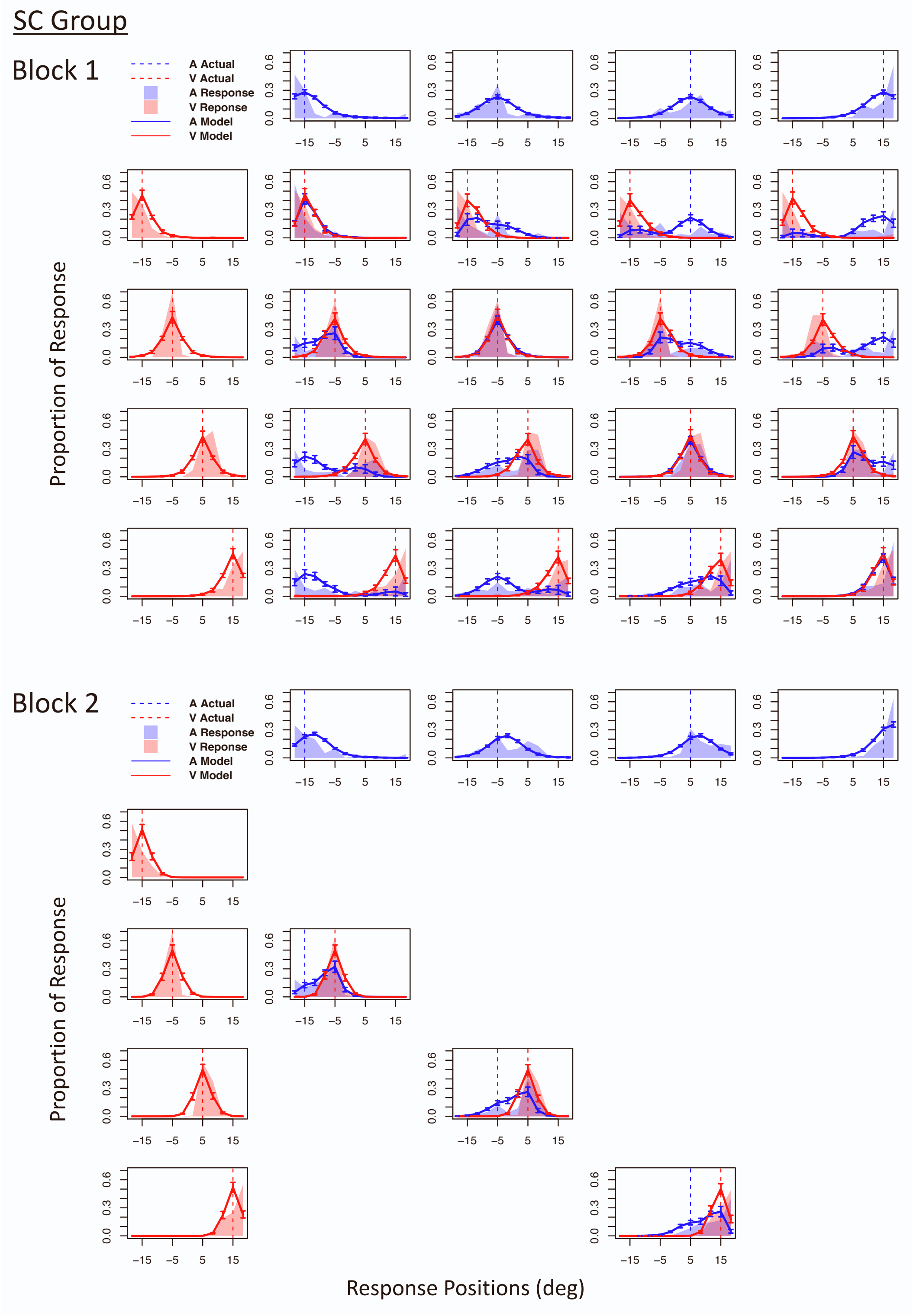

Figure S4. Auditory (A) and visual (V) localization response distributions and model best fits of Block 1 (VE/VAEi) and Block 2 (VAEc) for group SC. Related to Table 1, Figure 2, and Figure 5. Blue: A localization. Red: V localization. Vertical dotted lines indicate the actual stimulus locations. Shaded areas illustrate the proportion of responses at each response button position. Solid lines are the individually fitted model predictions (mean ± SEM), simulated using each participant's best-fitting model and parameters and then averaged within the group. In each sub-figure, the top row shows the unimodal A trials at four A locations, the left column shows the unimodal V trials at four V locations, and the remaining panels show the bimodal audiovisual (AV) trials with both A and V localization responses. In Block 2 (bottom), there are only three bimodal panels because the AV discrepancy was constant at +10°, yielding only three possible (A, V) location combinations: (-15°, -5°), (-5°, 5°), and (5°, 15°).

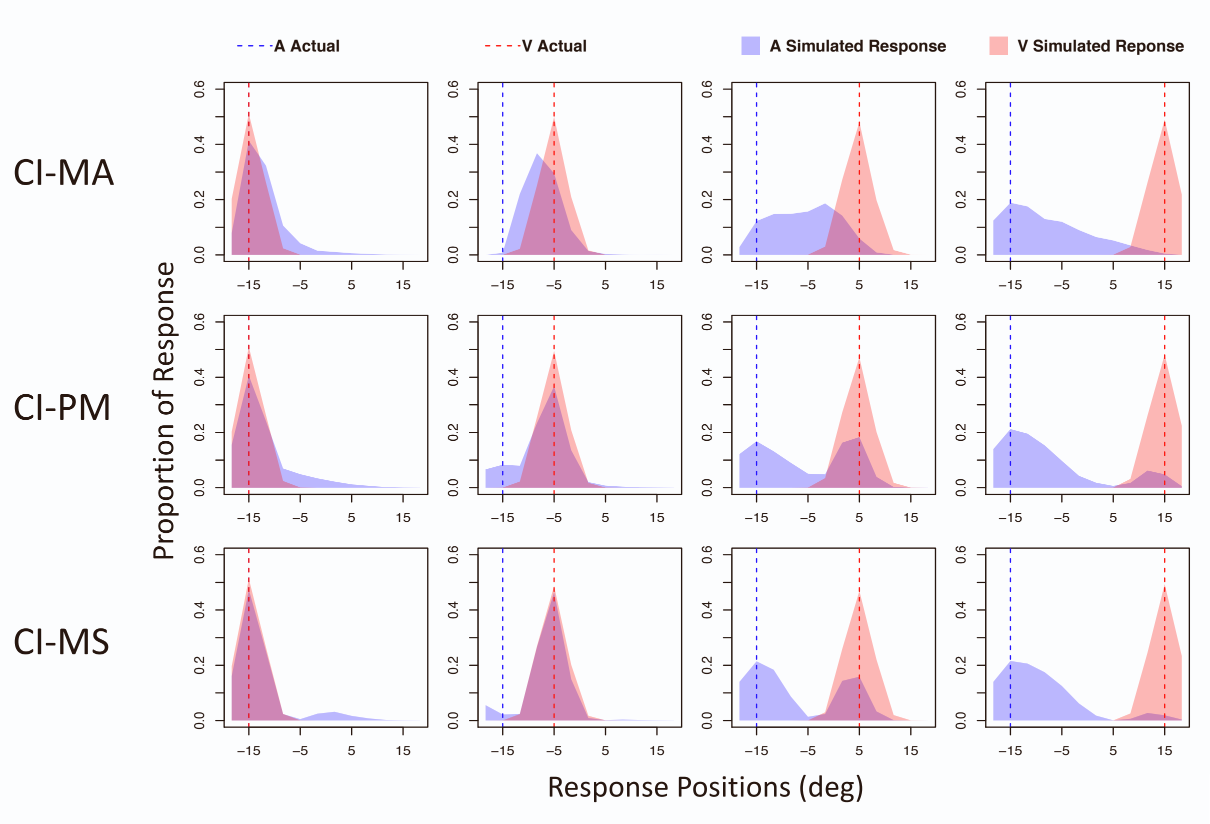

Figure S5. Simulated audiovisual localization response patterns for illustrating the different predictions of Bayesian causal inference (CI) models with different decision strategies. Related to Table 1 and STAR Methods. CI-MA: causal inference with model-averaging strategy. CI-PM: causal inference with probability-matching strategy. CI-MS: causal inference with model-selection strategy. Vertical dotted lines indicate the actual stimulus locations. Shaded areas depict the proportion of model-predicted responses at each response button position. Blue: auditory (A) localization. Red: visual (V) localization. The response distributions are generated via Monte Carlo simulations, with 10,000 simulated A responses and 10,000 simulated V responses for each pair of (A, V) locations, using the same parameters (Δ_A = 0, σ_A = 9, Δ_V = 0, σ_V = 2.5, μ_P = 0, σ_P = 40, Pc = 0.4) and changing only the decision strategy. For each decision strategy (row), four pairs of (A, V) locations are shown as examples (columns), with the A location always at -15°, and the V location at -15°, -5°, 5°, and 15° from the leftmost to the rightmost column, illustrating the increasing AV discrepancy. All three CI models predict similar V responses (all columns), fused audiovisual responses when A and V stimuli are at the same location (first column), largely fused responses when A and V stimuli are close (second column), largely segregated responses when A and V stimuli are distant (fourth column), and a transition from fusion to segregation of A and V cues as AV discrepancy increases (from first column to fourth column). The model predictions differ mainly in the A response pattern in the middle of this transition when the underlying causal structure is ambiguous (third column): CI-MA predicts smooth, typically unimodal combination of the fused and segregated A estimates, whereas CI-PM and CI-MS predict typically bimodal combinations of the fused and segregated A estimates. Compared with the CI-PM prediction, the bimodal distribution of A responses in the CI-MS prediction is more distinct, with a sharper boundary and less overlap between the two probability masses. In the bimodal A response distributions, the two modes are consistent with the A estimates obtained under a common cause (C = 1) and independent causes (C = 2), respectively; the probability mass around each mode varies with the model's estimated probability for each causal structure.

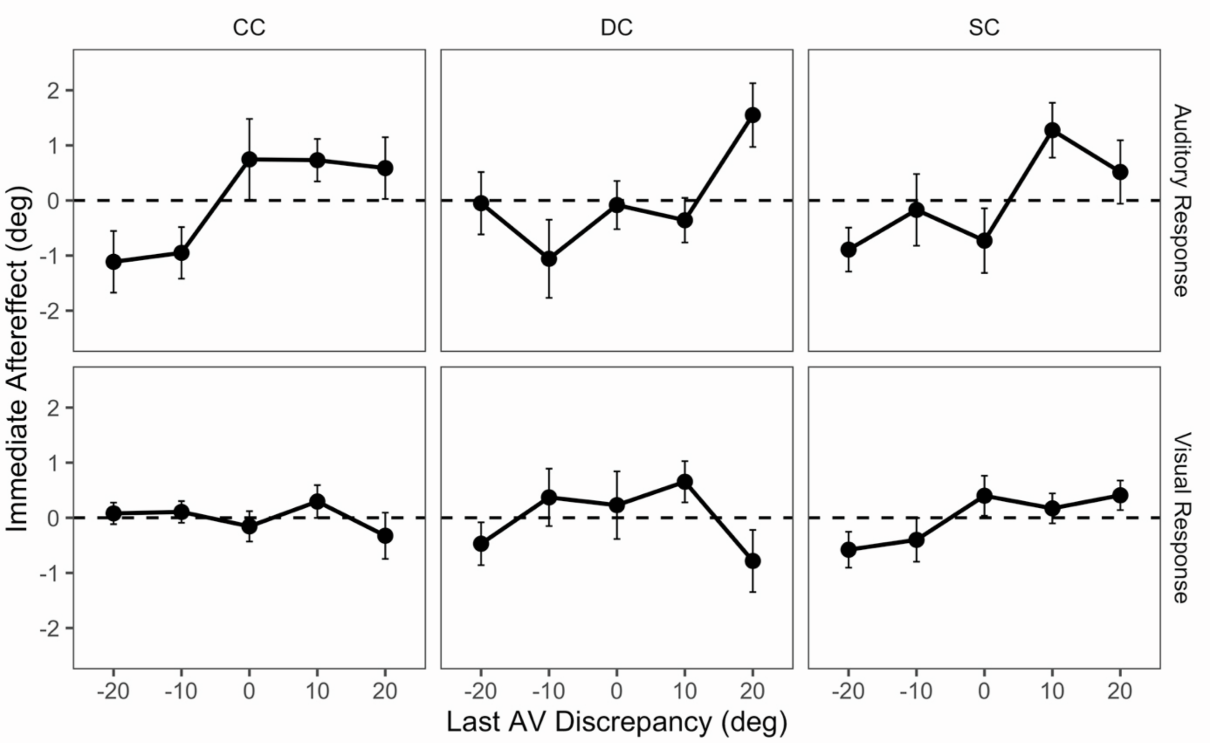

Figure S6. Mean localization responses (in degrees) in unimodal A and V trials as a function of AV spatial discrepancy in the preceding AV trial in CC, DC, and SC individuals. Related to Figure 3. Positive AV discrepancies indicate AV trials with a rightward spatial discrepancy (V to the right of A) and negative AV discrepancies indicate AV trials with a leftward discrepancy (V to the left of A). Positive *y*-axis values indicate rightward localization biases and negative values indicate leftward localization biases. Error bars indicate *SEM*s.

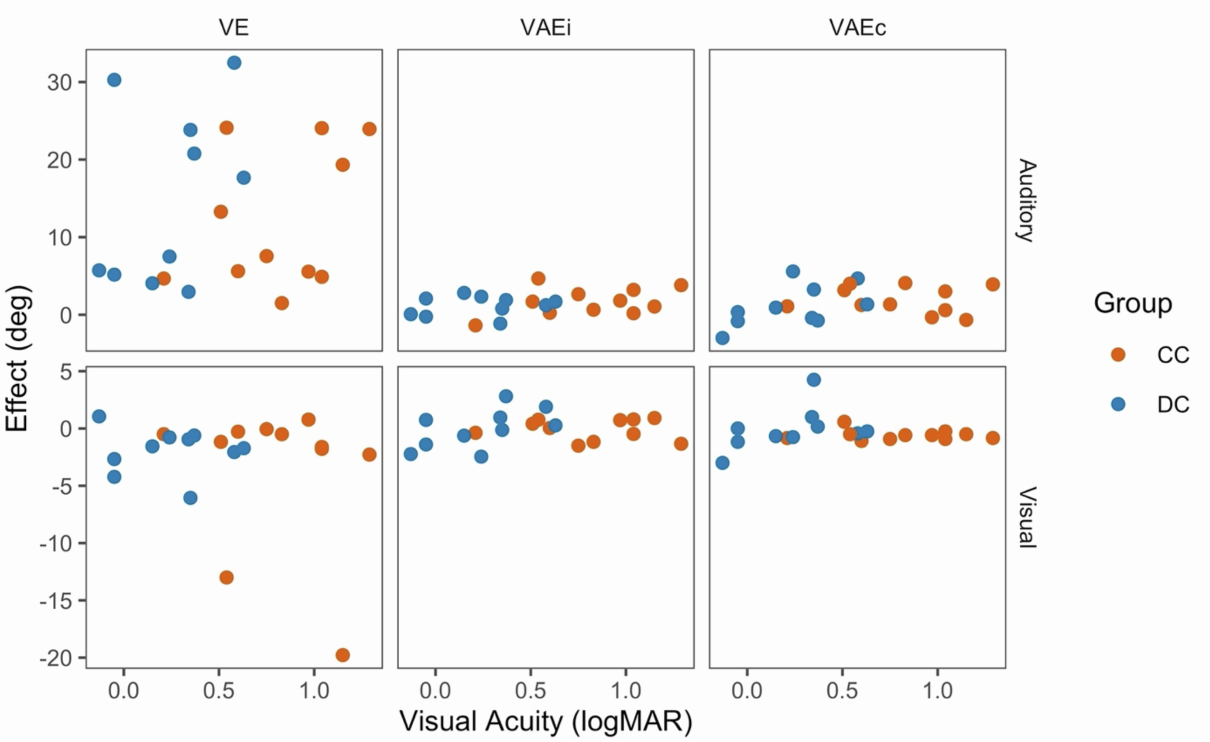

Figure S7. Correlations between visual acuity and VE (left panel), VAEi (middle panel), and VAEc (right panel). Related to Figure 2, Figure 3, and Figure 5. Scatterplots are shown separately for A and V effects. Colors indicate participant groups.

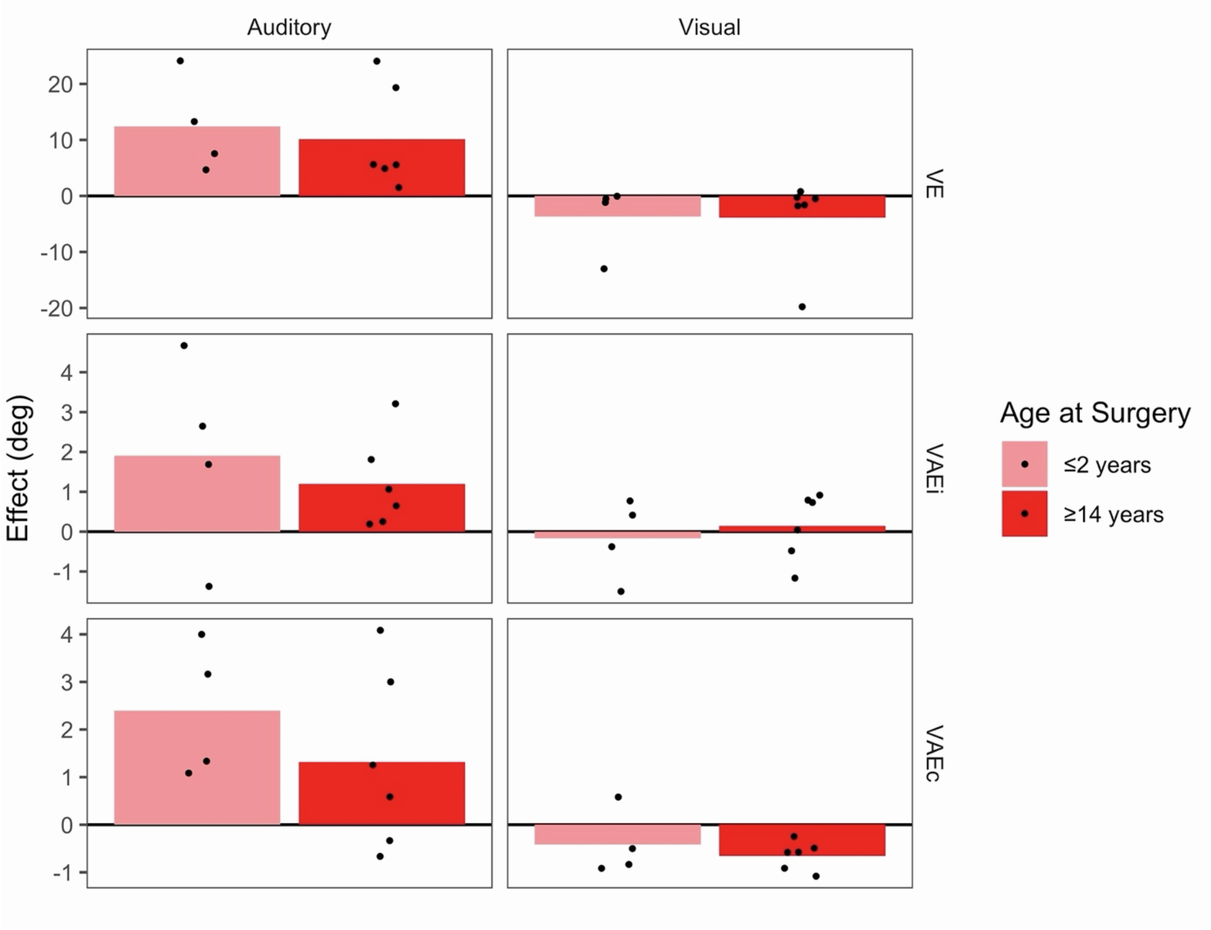

Figure S8. Ventriloquism effects (VE), immediate (VAEi) and cumulative (VAEc) ventriloquism aftereffects in CC individuals who had experienced short (≤2 years) versus long (≥14 years) periods of visual deprivation from birth. Related to Figure 2, Figure 3, and Figure 5. Bars indicate group averages and dots indicate individual values. Positive values indicate localization biases toward the A location and negative values indicate localization biases toward the V location. Note that two CC individuals who had presented for surgery with absorbed cataracts later in life (>14 years of age) did no longer fulfil the World Health Organization (2019) criteria for blindness (but still had severe vision impairment) at the time of surgery (see Table S3). However, although the exact age of cataract absorption in these two individuals is unknown to us, cataract absorption typically does not occur before middle childhood, suggesting that they had experienced an extended period of blindness (i.e., more than 2 years) from birth.
